# cAMP is an allosteric modulator of DNA-binding specificity in the cAMP receptor protein from *Mycobacterium tuberculosis*

**DOI:** 10.1016/j.jbc.2021.100480

**Published:** 2021-02-26

**Authors:** Fernanda Gárate, Stephen Dokas, Maria Fe Lanfranco, Clare Canavan, Irina Wang, John J. Correia, Rodrigo A. Maillard

**Affiliations:** 1 Department of Chemistry, Georgetown University, Washington, District of Columbia, USA; 2 Department of Cell and Molecular Biology, The University of Mississippi Medical Center, Jackson, Mississippi, USA

**Keywords:** allosteric regulation, cyclic AMP (cAMP), CRP, linkage, bacterial transcription, protein folding, structural homology, fluorescence anisotropy, ITC, MTB, ANS, 8-anilino-1-naphthalenesulfonic acid, AUC, analytical ultracentrifugation, BCG, Bacille Calmette-Guérin, *c*, cooperativity factor between cAMP-binding sites, CRP, cAMP receptor protein, CRP_BCG_, cAMP receptor protein from *Mycobacterium bovis* Bacille Calmette-Guérin strain, CRP_Ecoli_, cAMP receptor protein from *Escherichia coli*, CRP_MTB_, cAMP receptor protein from *Mycobacterium tuberculosis*, ΔG°, Gibbs free energy change, ITC, isothermal titration calorimetry, k_1_, cAMP-binding affinity constant for the first cAMP-binding site, k_2_, cAMP-binding affinity constant for the second cAMP-binding site, *K*_d_, dissociation constant, k_DNA_, DNA-binding affinity constant, *m*, m-value, MTB, *Mycobacterium tuberculosis*, SV, sedimentation velocity

## Abstract

Allosteric proteins with multiple subunits and ligand-binding sites are central in regulating biological signals. The cAMP receptor protein from *Mycobacterium tuberculosis* (CRP_MTB_) is a global regulator of transcription composed of two identical subunits, each one harboring structurally conserved cAMP- and DNA-binding sites. The mechanisms by which these four binding sites are allosterically coupled in CRP_MTB_ remain unclear. Here, we investigate the binding mechanism between CRP_MTB_ and cAMP, and the linkage between cAMP and DNA interactions. Using calorimetric and fluorescence-based assays, we find that cAMP binding is entropically driven and displays negative cooperativity. Fluorescence anisotropy experiments show that apo-CRP_MTB_ forms high-order CRP_MTB_–DNA oligomers through interactions with nonspecific DNA sequences or preformed CRP_MTB_–DNA complexes. Moreover, we find that cAMP prevents and reverses the formation of CRP_MTB_–DNA oligomers, reduces the affinity of CRP_MTB_ for nonspecific DNA sequences, and stabilizes a 1-to-1 CRP_MTB_–DNA complex, but does not increase the affinity for DNA like in the canonical CRP from *Escherichia coli* (CRP_Ecoli_). DNA-binding assays as a function of cAMP concentration indicate that one cAMP molecule per homodimer dissociates high-order CRP_MTB_–DNA oligomers into 1-to-1 complexes. These cAMP-mediated allosteric effects are lost in the double-mutant L47P/E178K found in CRP from *Mycobacterium bovis* Bacille Calmette-Guérin (CRP_BCG_). The functional behavior, thermodynamic stability, and dimerization constant of CRP_BCG_ are not due to additive effects of L47P and E178K, indicating long-range interactions between these two sites. Altogether, we provide a previously undescribed archetype of cAMP-mediated allosteric regulation that differs from CRP_Ecoli_, illustrating that structural homology does not imply allosteric homology.

Signal transduction is an essential process that allows cells to cope and respond to changes in their environment (1). Many signaling pathways rely on small molecules to transduce external stimuli to one or more effector proteins inside the cell (2). cAMP is an ancient, ubiquitous small molecule that serves as a second messenger in many signal transduction pathways, including regulation of gene expression in response to changes in environmental conditions (3, 4, 5).

The cAMP receptor protein (CRP) is a homodimeric transcription factor targeted by cAMP (6, 7, 8). Each CRP subunit harbors a structurally conserved N-terminal cAMP-binding domain that is covalently linked to a DNA-binding domain located in the C-terminal portion of the protein (9, 10, 11, 12, 13, 14). Solution biophysical and structural studies have shown that cAMP binding to the CRP from *Escherichia coli* (CRP_Ecoli_) stimulates a large conformational change in the DNA-binding domains (Fig. 1, *A* and *B*, *top*) (10, 13, 15, 16, 17). In contrast, structures of the CRP from *Mycobacterium tuberculosis* (CRP_MTB_) in the apo-form and cAMP-bound form reveal smaller cAMP-induced conformational changes (Fig. 1, *A* and *B*, *bottom*) (11, 14). The CRP_Ecoli_ and CRP_MTB_ display structural differences in both the apo-state and cAMP-bound state, most notably in their DNA-binding domain orientations relative to the cAMP-binding domains (Fig. 1*C*). Additional differences are observed in the homodimer symmetry. In the CRP_Ecoli_, the two subunits in the apo-state are symmetric, but the cAMP-bound state shows asymmetry between the DNA-binding domains' conformation (18) (Fig. 1*D*, *top*). Conversely, the subunits in the CRP_MTB_ in the apo-state are asymmetric at the dimer interface helix (c-helix) and the DNA-binding domains, but the cAMP-bound state is highly symmetric (Fig. 1*D*, *bottom*). Finally, the CRP_MTB_ harbors two additional α-helices, one at the N terminus and another at the C terminus, resulting in a slightly larger protein than the CRP_Ecoli_ (Fig. 1*E*).

**Figure 1:**
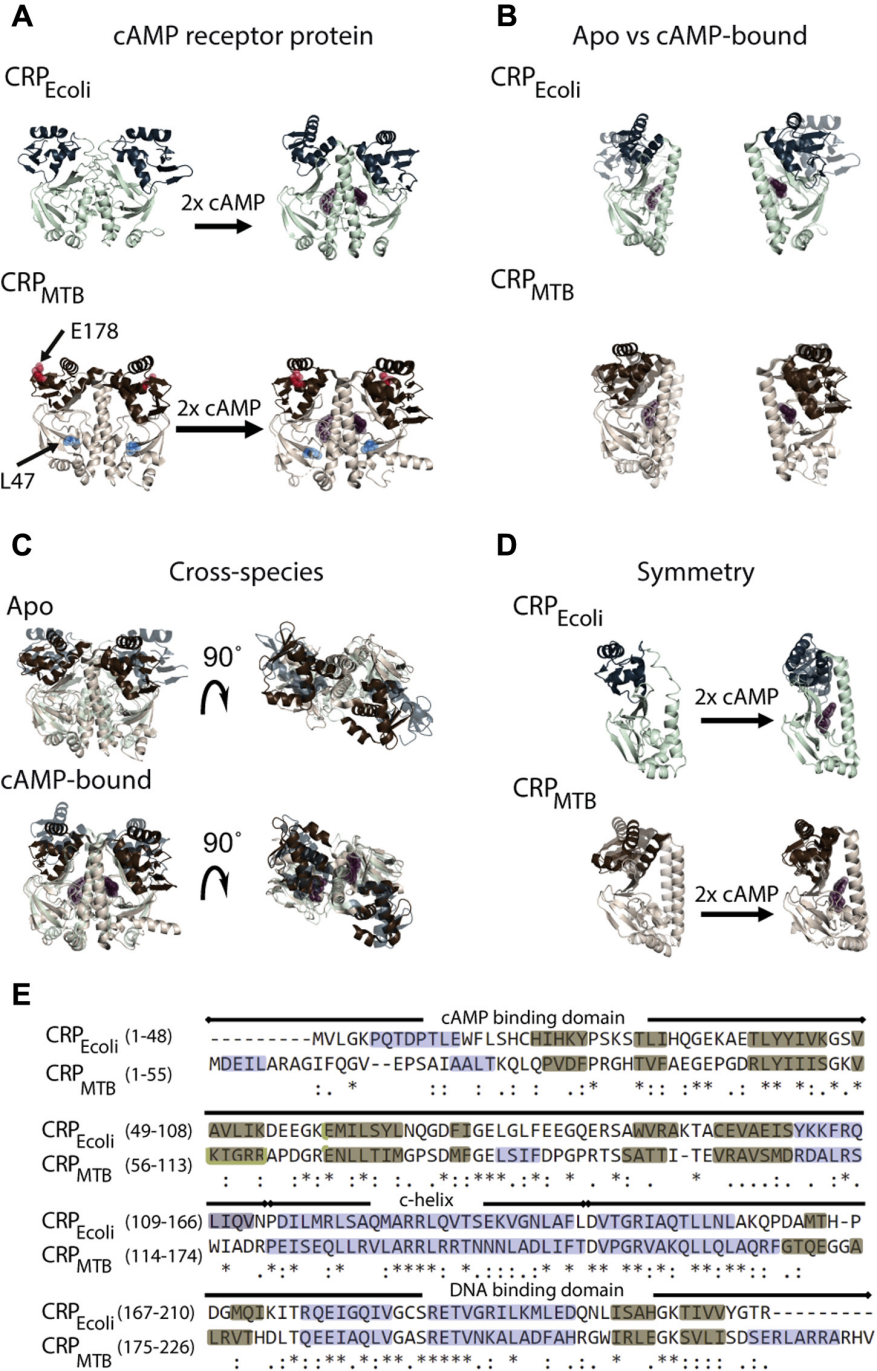
**Structural comparison between the CRP**_**Ecoli**_**and CRP**_**MTB**_. The CRP has a conserved structural organization with two identical subunits, each one harboring a cAMP-binding domain in the N terminus (*pale cyan* in the CRP_Ecoli_; *tan* in the CRP_MTB_) and a DNA-binding domain in the C terminus (*dark teal* in the CRP_Ecoli_; *dark brown* in the CRP_MTB_). *A*, structures of the CRP_Ecoli_ and CRP_MTB_ in the absence and presence of cAMP. *B*, alignment of apo-subunit (*light teal* and *light brown*) and cAMP-bound subunit for both the CRP_Ecoli_ (*top*) and CRP_MTB_ (*bottom*). *C*, alignment of the CRP_Ecoli_ and CRP_MTB_ homodimers. *D*, alignment of intraspecies monomers (*left*, apo-state; *right*, cAMP-bound state) for the CRP_Ecoli_ (*top*) and CRP_MTB_ (*bottom*). *E*, the CRP_Ecoli_ and CRP_MTB_ sequence alignment with mapped secondary structures (α-helices in *blue*; β-strands in *light brown*); (*asterisk* indicates residue identity; *colon* indicates similar residues, and *dot* indicates weakly similar residues). Differences in the sequence between the CRP_MTB_ and CRP_BCG_ are located at positions E178 (*red spheres*) and L47 (*light blue spheres*). cAMP is shown as *brown spheres*. See Experimental procedures for description of alignment and Protein Data Bank codes. CRP, cAMP receptor protein; CRP_Ecoli_, CRP from *Escherichia coli*; CRP_MTB_, CRP from *Mycobacterium tuberculosis*.

The CRP_Ecoli_ and CRP_MTB_ show structural differences, and their functional response to cAMP binding also differs. In the CRP_Ecoli_, cAMP binding enhances the affinity of the protein to tightly interact with pseudopalindromic gene promoter sequences involved in carbohydrate metabolism (18, 19, 20, 21, 22, 23). In the CRP_MTB_, the affinity for DNA promoters with and without cAMP appears to be similar (12, 20, 24). The small cAMP-induced conformational change in the CRP_MTB_ provides a structural explanation by which this protein does not change its affinity to DNA upon binding to the cyclic nucleotide (6, 12, 16, 25). It is therefore possible that the CRP_MTB_ is not sensitive to cAMP, but previous studies have shown that cAMP interactions with the CRP_MTB_ are important in the regulation of the gene *whiB1* (12, 26). It is therefore possible that the allosteric regulation triggered by cAMP in the CRP_MTB_ may not be directly associated to large changes in protein conformation that enhance the affinity for specific DNA promoter sequences, as seen in the CRP_Ecoli_. To dissect the mechanisms by which cAMP allosterically regulates CRP_MTB_–DNA binding, in this study, we quantitatively characterize the linkage between cAMP and DNA interactions. We combine complementary solution biophysical approaches to measure cAMP-binding affinity and cooperativity, interactions with the DNA promoter SerC (6, 27) as a function of cAMP concentration, and protein solution structure, assembly, and thermodynamic stability.

The results from this study reveal that the CRP_MTB_ binds cAMP with moderate negative cooperativity. In agreement with previous reports (12), the affinity of the CRP_MTB_ for promoter sequences is similar in the presence and in the absence of cAMP, indicating that the cyclic nucleotide does not regulate transcription at the level of affinities to specific DNA promoter sequences. We find that in the apo-state, the protein forms high-order CRP_MTB_–DNA oligomers. These oligomers are mediated by interactions between the CRP_MTB_ and nonspecific DNA sequences, and by interactions between a CRP_MTB_–DNA complex and the free CRP_MTB_. Unexpectedly, the presence of cAMP decreases nonspecific interactions with DNA and reversibly dissociates high-order CRP_MTB_–DNA oligomers into stable, 1-to-1 stoichiometric complexes. We also investigated the double-mutant L47P/E178K, which is found in the CRP from the attenuated *Mycobacterium bovis* Bacille Calmette-Guérin strain (CRP_BCG_) (mutation sites shown in Fig. 1*A*, *bottom*) and only differs from the CRP_MTB_ sequence in those two amino acid residues (28, 29, 30). While the CRP_BCG_ displays negative cAMP-binding cooperativity like the CRP_MTB_, we find that cAMP does not prevent the formation of high-order CRP_BCG_–DNA oligomers. These functional differences are not observed in the single mutants L47P (CRP_MTB_–L47P) and E178K (CRP_MTB_–E178K), indicating nonlinear contributions and long-range interactions between the two mutation sites. In agreement with nonlinear mutant contributions, the thermodynamic stability and dimerization constant of the CRP_BCG_ are also different from the single mutants.

In combination, these results provide an archetype of cAMP-mediated regulation that is significantly different from those described previously in other CRPs, such as the well-characterized *E. coli* homolog, and illustrate that structural homology does not imply allosteric homology. In other words, two structures could be very similar but respond very differently to the same allosteric effector.

## Results

### CRP_MTB_ exhibits negative cooperativity between the two cAMP-binding domains

**Figure 2:**
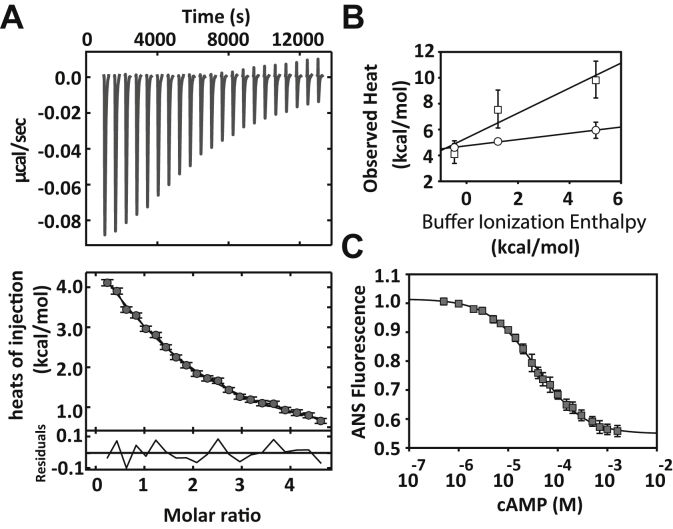
**Characterization of cAMP binding to the CRP**_**MTB**_. *A*, the *upper panel* corresponds to the calorimetry data of the titration of cAMP monitored by ITC in Hepes buffer. The *lower panel* shows the resulting cAMP-binding isotherm. The *solid line* represents the fit using a sequential two-site binding model with residuals (Equations (12), (14) in Experimental procedures). *B*, buffer ionization enthalpy for each cAMP-binding event: from *left* to *right*: cacodylate, PBS, and Hepes buffer. The error bars correspond to the SD from three to four experimental repeats. We find that ΔH_1_ in each buffer is not statistically indistinguishable (*p* = 0.23), but ΔH_2_ shows statistical pairwise differences among all buffers (*p* = 3 × 10^−4^). *C*, cAMP binding monitored by changes in ANS fluorescence. The *solid line* represents the fit using a two-site binding model (Equation 16 in Experimental procedures). CRP_MTB_, CRP from *Mycobacterium tuberculosis*; ITC, isothermal titration calorimetry; ΔH_1_, enthalpy change for the first cAMP-binding site; ΔH_2_, enthalpy change for the second cAMP-binding site; ANS, 8-anilino-1-naphthalenesulfonic acid.

We first used isothermal titration calorimetry (ITC) to quantitatively determine the cAMP-binding affinity constants, cooperativity, and their underlying thermodynamic driving forces (Fig. 2*A*, Table 1). To ensure full saturation of the CRP_MTB_, we conducted ITC experiments using up to a five-fold molar excess of cAMP to protein. By fitting the ITC data to various binding models, we found that a two-site sequential binding mechanism (19, 24) provided better fitting statistics than a set of independent binding sites (Fig. S1 and Table S1). The site-specific binding constants we obtained were *k*_1_ = (3.0 ± 0.9)⋅10^4^ M^−1^ and *k*_2_ = (1.2 ± 0.2)⋅10^4^ M^−1^ (all errors represent the SD of the fit). The ratio between the two binding constants, *c* = *k*_2_/*k*_1_ = 0.4 ± 0.2, indicates negative cooperativity between the two cAMP-binding domains (Table 1). ITC experiments using various buffers (Hepes, PBS, and cacodylate, Fig. S2 and Table S1) were performed to dissect a potential contribution of proton ionization to the observed cAMP-binding enthalpies and to determine whether the release or uptake of protons is associated with the cAMP-binding reactions (31, 32). We find that both cAMP-binding events are endothermic (enthalpy change for the first cAMP-binding site = 4.7 ± 0.3 kcal mol^−1^ and enthalpy change for the second cAMP-binding site = 5.0 ± 1.0 kcal mol^−1^) and therefore entropically driven (*T*Δ*S*_1_ = 10.8 kcal mol^−1^ and *T*Δ*S*_2_ = 10.5 kcal mol^−1^). Moreover, we find that the first cAMP-binding event is independent of the buffer-ionization enthalpy, whereas the second one displayed a slope of 1.0 ± 0.3, indicating proton uptake by the protein (Fig. 2*B*). The asymmetry in proton uptake during cAMP binding may be a consequence of the asymmetry seen in the apo-CRP_MTB_ structure (Fig. 1*D*, *bottom*) or asymmetric states in partially liganded conformations (19, 22). Importantly, in all three buffers used in this study, a two-site sequential binding mechanism resulted in better fitting statistics (Table S2) and revealed negative cAMP-binding cooperativity (Table 2). In addition to ITC experiments, we monitored cAMP binding *via* changes in 8-anilino-1-naphthalenesulfonic acid (ANS) fluorescence (Fig. 2*C*) (19, 24, 33). The data were fitted to two binding polynomials, wherein a model allowing for cooperativity provided a statistically better fit than a model with independent binding sites (Fig. S3 and Table S2). Moreover, the binding constants obtained from the ANS-based assay are in agreement with the results using ITC and support the observed negative cooperativity between the two cAMP-binding sites: *k*_1_ = (3.5 ± 0.2)⋅10^4^ M^−1^ and *k*_2_ = (2.2 ± 0.1)⋅10^4^ M^−1^ and *c* = 0.6 ± 0.1 (Table 1).

**Table 1:** cAMP-binding affinity constants for the CRP_MTB_

Method	cAMP-binding affinity		Binding cooperativity
	*k*_1_	*k*_2_	*c*
ITC	3.0 ± 0.9	1.2 ± 0.2	0.4 ± 0.2
ANS	3.5 ± 0.2	2.2 ± 0.1	0.6 ± 0.1

ANS, 8-anilino-1-naphthalenesulfonic acid; *c*, cooperativity factor between cAMP-binding sites; CRP_MTB_, CRP from *Mycobacterium tuberculosis*; ITC, isothermal titration calorimetry; *k*_1_, cAMP-binding affinity constant for the first cAMP-binding site; *k*_2_, cAMP-binding affinity constant for the second cAMP-binding site.

The error corresponds to the SD from fitted parameters using a two-site binding model as described in Experimental procedures. The units of *k*_1_ and *k*_2_ are 10^4^ M^−1^ and *c* = *k*_2_/*k*_1_.

**Table 2:** Buffer effect on cAMP-binding affinity constants for the CRP_MTB_ measured by ITC

Buffer	*k*_1_	*k*_2_	*c*
Cacodylate	3.9 ± 0.9	1.8 ± 0.4	0.45 ± 0.13
PBS	3.4 ± 0.5	1.2 ± 0.8	0.35 ± 0.13
Hepes	3.0 ± 0.9	1.2 ± 0.2	0.39 ± 0.11

*c*, cooperativity factor between cAMP-binding sites; CRP_MTB_, CRP from *Mycobacterium tuberculosis*; ITC, isothermal titration calorimetry; *k*_1_, cAMP-binding affinity constant for the first cAMP-binding site; *k*_2_,cAMP-binding affinity constant for the second cAMP-binding site.

The error corresponds to the SD from fitted parameters using a two-site binding model as described in Experimental procedures. The units of *k*_1_ and *k*_2_ are 10^4^ M^−1^ and *c* = *k*_2_/*k*_1_.

### CRP_MTB_–DNA interactions as a function of cAMP concentration

Previous structural studies have shown that binding of cAMP induces small conformational changes to the DNA-binding domain of the CRP_MTB_, allowing the protein to switch from an asymmetric structure to a symmetric, active conformation (11, 14) (Fig. 1*D*, *bottom*). However, the effect of this conformational transition on the affinity between the CRP_MTB_ and DNA promoter sequences is not fully understood. Thus, we investigated the role that cAMP occupancy plays in the formation of the CRP_MTB_–DNA complex. The formation of the CRP_MTB_–DNA complex was monitored *via* changes in fluorescence anisotropy, normalized to the first protein concentration point (19, 20). We used a 32-bp fluorescein-labeled SerC promoter, a well-characterized promoter targeted by the CRP_MTB_ (6, 14, 27, 34). These experiments were conducted using 0, 0.1, and 1 mM of cAMP. At these concentrations, the protein is in the apo-state, in a mix of singly and doubly cAMP-bound states (based on the binding affinity constants determined in this study) and in the doubly cAMP-bound state, respectively (Fig. S4).

**Figure 3:**
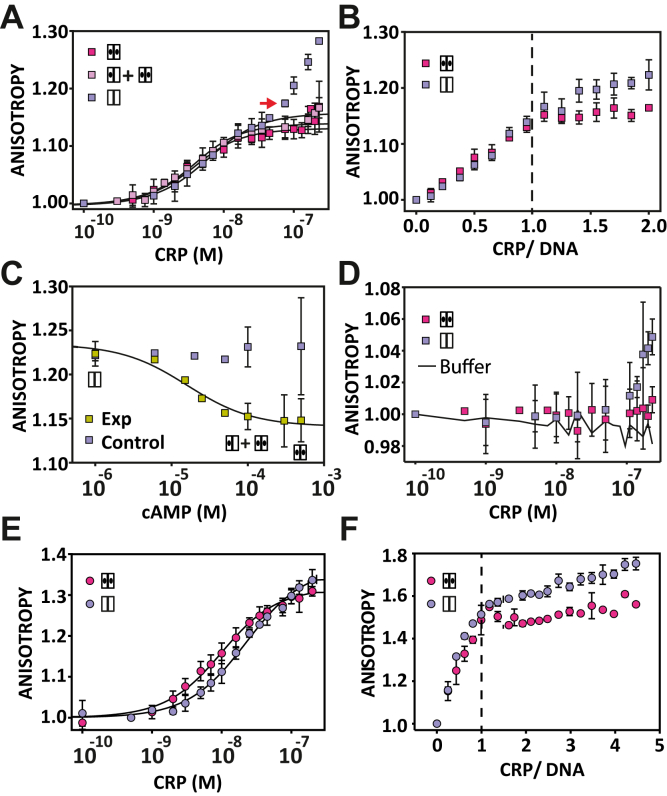
**Effect of cAMP on the CRP**_**MTB**_**interactions with the SerC promoter.***A*, CRP_MTB_–DNA complex formation using 3 nM of the 32-bp SerC promoter with cAMP concentrations equal to 0, 0.1, 1 mM (*light purple*, *light pink,* and *dark pink*, respectively). The *red arrow* indicates the titration point at which the anisotropy in the absence of cAMP significantly increases from experiments in its presence. *B*, stoichiometric binding using 200 nM of DNA (32-bp SerC) for the apo-conformation (*light purple*) and doubly cAMP-bound (*dark pink*) conformations. The *vertical dashed line* shows the CRP_MTB_–DNA complex formed at a 1-to-1 molar ratio. *C*, cAMP binding to the preformed CRP_MTB_–DNA complex using 230 nM of protein and 3 nM of 32-bp SerC promoter fragment. *Dark yellow* and *light purple squares* correspond to the cAMP titration and buffer titration (*i.e.*, control experiment), respectively. *D*, binding of the CRP_MTB_ to a 32-bp scramble sequence (3 nM). *Light purple* and *dark pink squares* correspond to the apo-conformation and doubly cAMP-bound conformation, respectively. The *solid line* corresponds to a control experiment with the buffer added instead of the protein. *E*, the CRP_MTB_–DNA complex formation using a 20-bp-long SerC promoter (3 nM). *Dark pink* and *light purple circles* correspond to the cAMP titration curve for the apo-conformation and doubly cAMP-bound conformations, respectively. *F*, stoichiometric binding using 400 nM of DNA (20-bp-long SerC) for the apo-conformation (*light purple*) and doubly cAMP-bound (*dark pink*) conformations. The *dashed line* denotes the concentration of the CRP by which the formation of the CRP_MTB_–DNA complex is at a 1-to-1 molar ratio. *Solid lines* in panels A and E are the fit as described in Equation 18 in Experimental procedures. In all panels, error bars correspond to the SD of 3 to 5 experimental repeats. CRP_MTB_, CRP from *Mycobacterium tuberculosis*; CRP, cAMP receptor protein.

In all three cAMP concentrations, the anisotropy of the labeled promoter increased as a function of the protein concentration, indicating that the formation of the CRP_MTB_–DNA complex occurs even in the absence of cAMP (Fig. 3*A*), a result that is in agreement with Rickman *et al*. (25) and Bai *et al*. (6). The DNA binding constants for the apo-state and doubly cAMP-bound state are *k*_DNA(apo)_ = (2.3 ± 0.9)⋅10^8^ M^−1^ and *k*_DNA(cAMP-2)_ = (4.2 ± 1.7)⋅10^8^ M^−1^, respectively (Table 3). At intermediate concentrations of cAMP (0.1 mM), where populations of singly and doubly cAMP-bound states coexist, we obtained similar binding affinities as in conditions used where only the doubly cAMP-bound conformation is populated. Altogether, these results indicate that the allosteric linkage initiated by cAMP binding is not associated with enhancing the binding affinity for specific DNA promoter sequences.

**Table 3:** SerC promoter binding affinity constants to the CRP_MTB_

Promoter length	DNA-binding affinity		
	*k*_DNA(apo)_	*k*_DNA(cAMP)_	*k*_DNA(cAMP-2)_
32-bp SerC promoter	2.3 ± 0.9	4.5 ± 1.2	4.2 ± 1.7
20-bp SerC promoter	0.12 ± 0.02	—	0.5 ± 0.1

CRP_MTB_, CRP from *Mycobacterium tuberculosis*; k_DNA(apo)_, DNA-binding affinity constant in the apo conformation; k_DNA(cAMP-2)_, DNA-binding affinity constant in the doubly cAMP-bound state; *k*_DNA(cAMP)_, DNA-binding affinity constant in the singly cAMP-bound state.

The error corresponds to the SD from fitted parameters as described in Experimental procedures.

The units of *k*_DNA(apo)_, *k*_DNA(cAMP)_, and *k*_DNA(cAMP-2)_ are 10^8^ M^−1^.

### cAMP prevents the formation of high-order CRP_MTB_–DNA oligomers

While the DNA-binding affinity constants were similar in all three concentrations of cAMP, we did observe important differences in the anisotropy signal toward the end of the titration (Fig. 3*A*, *red arrow*). In the absence of cAMP, the anisotropy signal gradually increased after the DNA-binding transition. In contrast, in the presence of cAMP (0.1 or 1 mM), the anisotropy signal remained nearly constant after the DNA-binding transition. This distinctive behavior suggests the formation of high-order CRP_MTB_–DNA oligomers in the apo-state that are prevented or reduced when the protein is bound to cAMP.

We confirmed these results by conducting stoichiometric DNA-binding assays using a concentration of the SerC promoter that is 10 to 20 times larger than the dissociation constant (*K*_d_) (Table 3). These experiments revealed a linear increase in the anisotropy signal that plateaus at a 1-to-1 molar ratio of protein to DNA, demonstrating that one molecule of the CRP_MTB_ binds to one molecule of DNA (Fig. 3*B*). In the absence of cAMP, we observe an overlap with the titration curve obtained with cAMP until a protein-to-DNA molar ratio of 1. However, after the 1-to-1 molar ratio is reached, the anisotropy signal in the apo-state continues to rise steadily, indicating again the formation of high-order CRP_MTB_–DNA oligomers.

In agreement with previous reports (12), our results show that cAMP does not have a large effect on DNA-binding affinities. However, titrations with a molar excess of protein to DNA, either with or without the cyclic nucleotide, suggest a noncanonical role for cAMP in allosteric signaling. Namely, that cAMP binding to the CRP_MTB_ prevents the formation of high-order DNA–protein oligomers. We therefore sought to determine what intermolecular interactions are involved in the formation of these oligomers and how cAMP binding prevents their formation.

### cAMP reverses preformed CRP_MTB_–DNA oligomers

Our previous experiments show that cAMP prevents the formation of high-order CRP_MTB_–DNA oligomers, yet to be determined is whether cAMP can reversibly dissociate such oligomers in a preformed state. To address this question, we preformed high-order CRP_MTB_–DNA oligomers and monitored changes in anisotropy as a function of the cAMP concentration (Fig. 3*C*). In these experiments, we used a concentration of CRP_MTB_ = 230 nM and the 32-bp fluorescein-labeled SerC promoter = 3 nM. At these concentrations of protein and DNA, we obtained the highest normalized anisotropy value that is experimentally accessible in the absence of cAMP, around 1.25 (Fig. 3*A*).

Figure 3*C* shows that upon titration of cAMP, the anisotropy signal of preformed CRP_MTB_–DNA oligomers decreases systematically, whereas in the absence of cAMP, the anisotropy remained constant. Importantly, the change in normalized anisotropy (∼0.09) upon cAMP binding is consistent with the difference in the normalized anisotropy signals seen between titration curves of the protein in the apo-state (∼1.25) and cAMP-bound states (∼1.15) (Fig. 3*A*). This quantitative agreement indicates that the decrease in anisotropy during the titration of cAMP corresponds to the reversible dissociation of high-order CRP_MTB_–DNA oligomers into a 1-to-1 complex. Furthermore, we fitted the changes in anisotropy as a function of cAMP to a single-site binding isotherm, which reflects the affinity of the preformed CRP_MTB_–DNA complex for cAMP. The apparent binding affinity constant was (6.3 ± 1.5)⋅10^4^ M^−1^, a value that is three times higher than the affinity of the first cAMP-binding site in the absence of DNA (Table 1). A two-site binding model did not improve the residuals of the fit (data not shown), suggesting that only one cAMP molecule per CRP dimer is sufficient to reversibly dissociate high-order CRP_MTB_–DNA oligomers. Given that apo-CRP_MTB_ binds cAMP with modest negative cooperativity, it is possible that the anisotropy assay cannot detect small differences in affinity between one or two cAMP-binding events.

### CRP_MTB_ binds nonspecifically to DNA in the absence of cAMP

To begin uncovering the molecular interactions that stabilize high-order CRP_MTB_–DNA oligomers, we first studied nonspecific DNA interactions using a 32-bp fluorescein-labeled scramble sequence. Because the scramble sequence lacks the conserved SerC-binding site (6), any changes in anisotropy would reflect nonspecific binding of the CRP_MTB_ to DNA. Figure 3*D* shows the titration curve of the CRP_MTB_ to the 32-bp scramble DNA sequence in the absence and presence of 1 mM cAMP. The titration shows identical anisotropy values in both conditions (*i.e.*, apo-state and cAMP-bound state) up to a concentration of protein of ∼80 nM. At higher protein concentrations, we observe an increase in anisotropy values only in the absence of cAMP, indicating that the protein in the apo-state is binding to DNA in a nonspecific manner. The control titration (solid black line, Fig. 3*D*), where only the buffer was added instead of the protein, shows negligible changes in anisotropy. These results suggest that the formation of high-order CRP_MTB_–DNA oligomers in the absence of cAMP can be driven by interactions with nonspecific DNA sequences.

### Formation of CRP_MTB_–DNA complexes with shorter promoter sequences

Next, we explored the nature of nonspecific DNA binding. We reasoned that the increase in anisotropy fluorescence in the absence of cAMP could arise from (1) apo-proteins interacting nonspecifically to flanking sequences outside the DNA footprint region or (2) binding of free proteins to preformed CRP_MTB_–DNA complexes. To distinguish between these two scenarios, we used SerC promoter sequences of decreasing lengths, down to the DNA footprint of the CRP_MTB_ based on the high-resolution structure, ∼18 bp (11, 14).

First, we determined the shortest DNA fragment that stably binds to the CRP_MTB_. We performed EMSAs using six different lengths of the SerC promoter (18, 20, 22, 24, 26, and 32 bps) (Fig. S5). Our data show that 20-bp is the minimum base pair length required for the CRP_MTB_ to bind robustly to DNA. A 20-bp SerC promoter sequence would only have 1-bp of overhang on each side, thereby minimizing potential protein interactions to DNA flanking regions.

The DNA binding constant for the 20-bp SerC promoter was quantitatively determined by fluorescence anisotropy. Figure 3*E* shows the titration curve of the CRP_MTB_ in the absence and presence of 1 mM cAMP. The DNA binding constants for the apo-state and cAMP-bound state were *k*_DNA(*apo*)_ = (0.12 ± 0.02)⋅10^8^ M^−1^ and *k*_DNA(cAMP-2)_ = (0.5 ± 0.1)⋅10^8^ M^−1^, respectively. These values are ∼10-fold lower than the binding constant for the 32-bp-long SerC promoter (Table 3). Importantly, stoichiometric binding assays shown in Figure 3*F* demonstrate that even in the almost complete absence of DNA flanking regions, there is still formation of high-order CRP_MTB_–DNA oligomers when cAMP is absent. We interpret this result as the free CRP_MTB_ binding to preformed CRP_MTB_–DNA complexes. Together with our previous results using the 32-bp scramble DNA sequence (Fig. 3*D*), our data indicate that these oligomers can be mediated by both nonspecific interactions between the protein and DNA and the binding of the free CRP_MTB_ to a preformed CRP_MTB_–DNA complex.

### Effect of mutations L47P and E178K on cAMP-binding affinity and cooperativity

The CRP_BCG_ only differs in two amino acids at positions L47P and E178K relative to the CRP_MTB_, which are located in the cAMP-binding domain and the DNA-binding domain, respectively (Fig. 1*A*, *bottom*). These mutations, which are not present in other CRP orthologs found in *M. bovis*, *M. tuberculosis*, or *Mycobacterium leprae*, have been implicated as potential contributing factors to the attenuation of BCG strains (28, 30). However, it remains unclear how the CRP_BCG_ differs from the CRP_MTB_ in its interaction mechanisms with cAMP or what are the contributions of each individual mutation toward cAMP-binding affinities and cooperativity.

**Figure 4:**
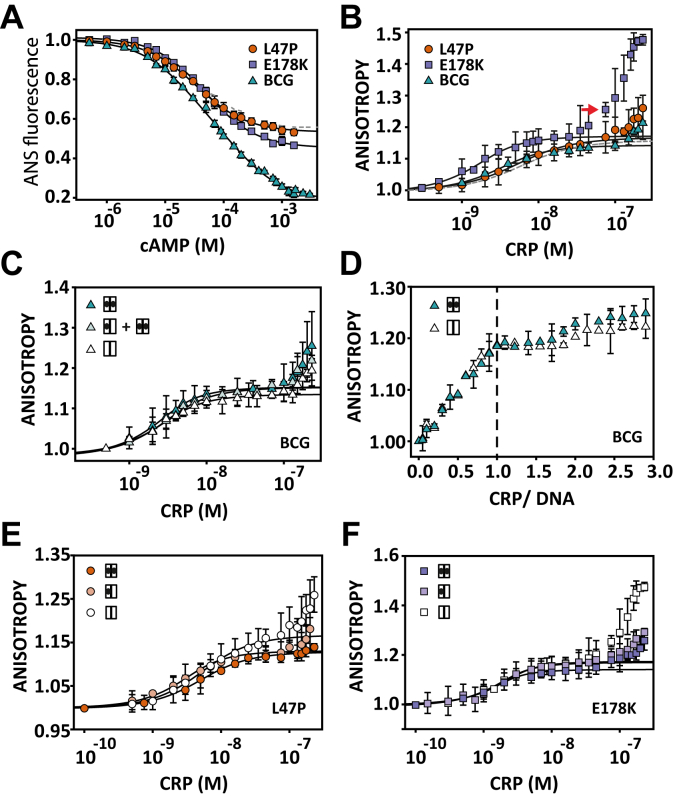
**Characterization of cAMP and DNA binding for the CRP**_**BCG**_**and single mutants CRP**_**MTB**_**–L47P and CRP**_**MTB**_**–E178K.***A*, cAMP binding monitored by changes in ANS fluorescence. The *solid line* corresponds to the fit using a two-site binding model (Equation 16 in Experimental procedures). *B*, DNA binding monitored by changes in anisotropy using 3 nM of the 32-bp fluorescein-labeled SerC promoter without cAMP. For comparison, the *dashed lines* in panels A and B correspond to the data of the CRP_MTB_. The *red arrow* indicates the titration point at which the anisotropy in the absence of cAMP significantly increases from experiments in its presence. *C*, binding of the CRP_BCG_ to the 32-bp fluorescein-labeled SerC promoter using 3 nM of DNA and three cAMP concentrations, 0, 0.1, 1 mM (*empty symbols*, *light green*, and *dark green*, respectively). *D*, stoichiometric binding of 32-bp fluorescein-labeled SerC promoter (200 nM) to the CRP_BCG_ in the apo-conformation (*empty symbols*) and doubly cAMP-bound conformation (*dark green*). The *dashed line* corresponds to the formation of the complex at a 1-to-1 molar ratio. Binding of CRP_MTB_–L47P (*E*) and CRP_MTB_–E178K (*F*) to the 32-bp fluorescein-labeled SerC promoter using 3 nM of DNA and three cAMP concentrations = 0, 0.1, 1 mM (*empty*, *light*, and *dark colored symbols*, respectively). Error bars in all panels correspond to the SD of at least 4 to 6 repeats. The *solid line* in panels *B*, *C*, *E*, and *F* corresponds to the fit using Equation 18 in Experimental procedures. CRP_BCG_, cAMP receptor protein from *Mycobacterium bovis* Bacille Calmette-Guérin strain; CRP_MTB_, CRP from *Mycobacterium tuberculosis.*

To answer these questions, we placed the individual mutations on the CRP_MTB_ (termed CRP_MTB_–L47P and CRP_MTB_–E178K) or the two together (CRP_BCG_) and determined their cAMP-binding affinities and cooperativity by monitoring changes in ANS fluorescence. Our data show that the affinity constants for the first cAMP-binding site (*k*_1_) are similar among the three CRP mutants, but the affinity for the second site (*k*_2_) was significantly lower for the CRP_BCG_ (Fig. 4*A*, Table 4). As a result, the cAMP-binding cooperativity ranges from neutral for CRP_MTB_–E178K and CRP_MTB_–L47P (*c* = 1) to negative for the CRP_BCG_ (*c* = 0.3) (Fig. S6). These results suggest that the cAMP-binding mode of the CRP_BCG_ is not attributed to a single mutation or a simple linear addition between the effects of the two individual mutations.

**Table 4:** cAMP- and DNA-binding affinity constants to the CRP_MTB_–L47P, CRP_MTB_–E178K, and CRP_BCG_

CRP mutant	cAMP-binding affinity and binding cooperativity			DNA-binding affinity		
	*k*_1_	*k*_2_	c	*k*_DNA(apo)_	*k*_DNA(cAMP)_	*k*_DNA(cAMP-2)_
L47P	2.9 ± 0.1	2.9 ± 0.1	1	2.7 ± 0.9	7.3 ± 3.0	2.9 ± 0.7
E178K	2.0 ± 0.1	2.0 ± 0.1	1	15.0 ± 5.3	10.0 ± 5.0	21.0 ± 12.9
BCG^a^	2.6 ± 0.7	0.9 ± 0.2	0.3 ± 0.2	5.6 ± 3.0	6.0 ± 5.3	7.5 ± 3.4

*c*, cooperativity factor between cAMP-binding sites; CRP_BCG_, cAMP receptor protein from *Mycobacterium bovis* Bacille Calmette-Guérin strain; CRP_MTB_, CRP from *Mycobacterium tuberculosis*; *k*_1_, cAMP-binding affinity constant for the first cAMP-binding site; *k*_2_, cAMP-binding affinity constant for the second cAMP-binding site; k_DNA(apo)_, DNA-binding affinity constant in the apo conformation; k_DNA(cAMP-2)_, DNA-binding affinity constant in the doubly cAMP-bound state; *k*_DNA(cAMP)_, DNA-binding affinity constant in the singly cAMP-bound state.

The error corresponds to the SD from fitted parameters as described in Experimental procedures. The units of *k*_1_ and *k*_2_ are 10^4^ M^−1^ and *c* = *k*_2_/*k*_1_. The units of *k*_DNA(apo)_, *k*_DNA(cAMP)_, and *k*_DNA(cAMP-2)_ are 10^8^ M^−1^.

a Because the CRP_BCG_ is in equilibrium between monomer and dimers, the reported affinities for DNA represent apparent binding affinity constants.

### Nonlinear effects of BCG mutations on CRP–DNA interactions

Given the nonlinear contributions of the individual BCG mutations over cAMP binding, we investigated the role of each mutation on DNA interactions using the 32-bp fluorescein-labeled SerC promoter. In the absence of cAMP, we found that CRP_MTB_–L47P has a DNA-binding affinity similar to that of the CRP_MTB_. However, CRP_MTB_–E178K binds to the promoter sequence with a ∼10-fold enhancement (Fig. 4*B*, Table 4). Because E178K is located at or is near the DNA-interaction surface (Fig. 1, *bottom*) and the mutation involves a change from a negatively to a positively charged amino acid side chain, it was not unexpected to observe a higher DNA-binding affinity than the CRP_MTB_ or CRP_MTB_–L47P. The unexpected result was that the CRP_BCG_ binds DNA with an affinity similar to that of the CRP_MTB_ or CRP_MTB_–L47P, indicating that the enhancing DNA-binding affinity effect of E178K is largely reduced by the presence of L47P.

CRP_MTB_–L47P and the CRP_BCG_ revealed an important difference in the formation of high-order CRP–DNA oligomers. In the absence of cAMP, both proteins did not form oligomers as pronouncedly as the CRP_MTB_ and CRP_MTB_–E178K. For example, at a concentration of the CRP_MTB_ of ∼50 nM (with [DNA] = 3 nM) the presence of oligomers becomes very evident and pronounced for the CRP_MTB_ and CRP_MTB_–E178K (red arrow in Figs. 3*A* and 4*B*, respectively). Neither CRP_MTB_–L47P nor the CRP_BCG_ forms noticeable CRP–DNA oligomers (Fig. 4*B*). These results again highlight nonlinear contributions of each BCG mutation toward both DNA-binding affinities and reduction in the formation of high-order CRP–DNA complexes. By comparison, the functional phenotype of the CRP_BCG_ is dominated by the contributions of the L47P mutation. Interestingly, the location of L47P is in the cAMP-binding domain, but its dominant effect over DNA interactions indicates long-range allosteric communication between cAMP- and DNA-binding domains.

### Effect of cAMP on CRP_BCG_–DNA interactions

We showed that the CRP_MTB_ and CRP_BCG_ have similar cAMP-binding affinity constants and display negative cooperativity (Table 1, Table 4). Here, we examined the linkage between cAMP and DNA binding for the CRP_BCG_. We monitored changes in anisotropy upon the formation of the CRP_BCG_–DNA complex (using the 32-bp fluorescein-labeled SerC promoter) at three cAMP concentrations: 0, 0.1, and 1 mM. At [cAMP] = 0.1 mM, 60% of the CRP_BCG_ population corresponds to the singly bound conformation, whereas the other 40% corresponds to doubly bound conformation. At 1 mM of cAMP, 90% of the population corresponds to a doubly cAMP-bound state, thus almost reaching a saturated state (Fig. S7). The anisotropy data revealed indistinguishable DNA-binding constants in all cAMP concentrations: *k*_DNA(*apo*)_ = (5.6 ± 3.0)·10^8^ M^−1^, DNA-binding affinity constant in the singly cAMP-bound state = (6.0 ± 5.3)·10^8^ M^−1^, and *k*_DNA(*cAMP*-*2*)_ = (7.5 ± 3.4)·10^8^ M^−1^ (Fig. 4*C*, Table 4). This result is consistent with titrations with the CRP_MTB_ that shows small cAMP effects over the interaction with the specific promoter-binding site. In contrast with the CRP_MTB_, the effect of cAMP binding in reducing high-order CRP-DNA oligomers was negligible for the CRP_BCG_. This was also observed in stoichiometric binding assays (Fig. 4*D*). We therefore explored the effect of cAMP on DNA interactions for each individual mutant (Fig. 4, *E* and *F*). We find that both the CRP_MTB_–L47P and CRP_MTB_–E178K form high-order CRP–DNA oligomers in the absence of cAMP, which are significantly reduced in the presence of intermediate (0.1 mM) or saturating (1 mM) amounts of cAMP (Fig. 4, *E* and *F*). Altogether, our DNA binding data are consistent with cAMP-binding studies that indicate asymmetric contributions of individual mutations to the CRP_BCG_ homolog. In this case, their influence on DNA interactions (specific or nonspecific) does not follow a simple linear combination, an analogous observation to results obtained in cAMP-binding assays (Fig. 4*A*).

### Solution structure and stability of the CRP_BCG_ differs from single mutants and the CRP_MTB_

**Figure 5:**
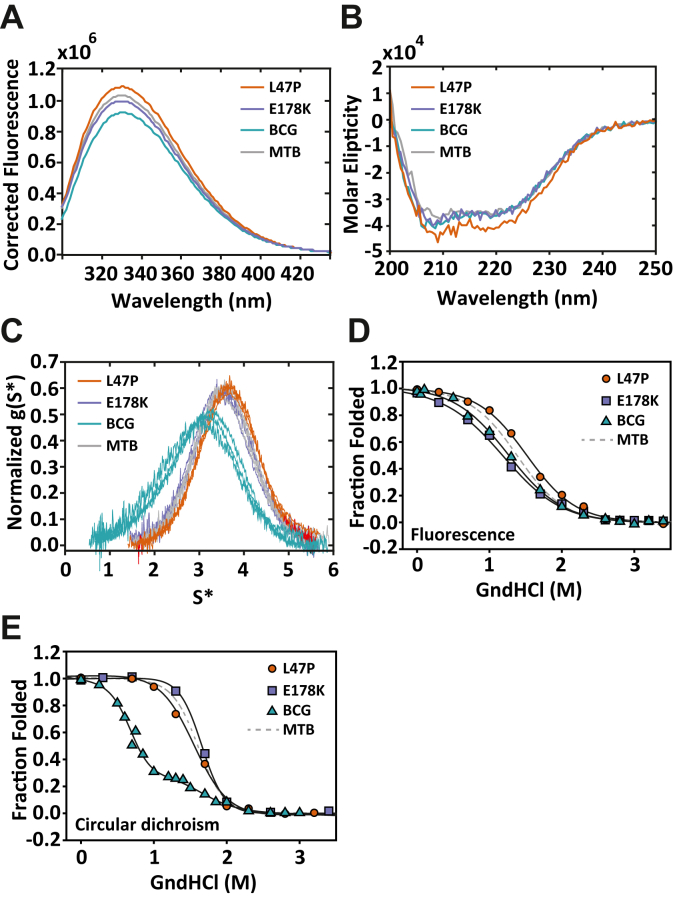
**Biophysical characterization of CRPs.***A*, tryptophan emission spectra of CRPs (5 mM) with an excitation wavelength of 295 nm. *B*, CD spectra of CRPs (5 mM). *C*, g(s) plots for CRPs showing concentration dependence for the CRP_BCG_. *Solid gray lines* in panels *A*–*C* represent the data for the CRP_MTB_ (labeled MTB). Chemical denaturation monitored by changes in tryptophan fluorescence (*D*) and CD (*E*). For comparison, *gray dashed lines* correspond to the CRP_MTB_ (labeled MTB). The *solid lines* are the fits using a two-state unfolding model for the individual mutants and a three-state unfolding model for the CRP_BCG_ (Equations (6), (11) in Experimental procedures, respectively). CRP_MTB_, CRP from *Mycobacterium tuberculosis*; CRP, cAMP receptor protein; CRP_BCG_, cAMP receptor protein from *Mycobacterium bovis* Bacille Calmette-Guérin strain; MTB, *Mycobacterium tuberculosis.*

The functional differences observed between the CRP_BCG_ and the single mutants CRP_MTB_–L47P and CRP_MTB_–E178K or the CRP_MTB_ may arise from differences in their native solution structure, assembly state, or stability. The protein solution structure and assembly were evaluated by using three biophysical methods: intrinsic protein fluorescence, CD, and analytical ultracentrifugation (AUC). The intrinsic fluorescence emission spectra were similar for all four CRPs, indicating that the tertiary structures surrounding the tryptophan residues (with excitation wavelength at 295 nm) are largely unaffected by the mutations (Fig. 5*A*). Spectra obtained using an excitation wavelength of 280 nm that includes the contribution of one tyrosine residue per subunit show no differences between all four CRPs (data not shown). Similarly, the CD spectra for all CRPs overlapped, indicating that the global native fold and secondary structure content of the proteins are the same (Fig. 5*B*).

The degree to which CRP is a stable homodimer was assessed by AUC (*i.e.*, sedimentation velocity [SV]). Experiments conducted at monomer concentrations ranging between 1 and 40 μM showed, with exception of the CRP_BCG_, a constant sedimentation coefficient corresponding to the molecular mass of the homodimer (52.2 kDa) and a dimerization *K*_d_ lower bound of 10 nM. This result indicates that the CRP_MTB_, CRP_MTB_–L47P, and CRP_MTB_–E178K were stable homodimers at concentrations of protein used throughout these studies (Fig. 5*C*). In contrast, the CRP_BCG_ showed monomer–dimer association processes (Fig. 5*C*, *cyan*). Nonlinear square fitting of the SV data indicate that the double mutant has a significantly lower dimerization *K*_d_ of ∼17.5 μM. This result suggests that the CRP_BCG_ was in a monomeric state in the DNA-binding assays conducted with a protein concentration in the nanomolar range. However, stoichiometric DNA-binding data (Fig. 4*D*) show a plateau when the DNA and CRP_BCG_ dimer concentrations reach a 1-to-1 ratio. This suggests that at equilibrium each CRP_BCG_ dimer forms a stable 1-to-1 complex with DNA. The alternative scenario, in which the CRP_BCG_ monomers were to form stable complexes with DNA, would reach a plateau in stoichiometric DNA-binding assays at molar ratios lower than 1. Thus, our results suggest that the CRP_BCG_ readily dimerizes when it binds DNA, but the dissection and quantification of the linkage between CRP_BCG_ dimerization and DNA interactions (with and without cAMP) remains unknown and is currently being investigated.

To determine mutational effects on protein stability, we monitored changes in tryptophan fluorescence (Fig. 5*D*) and CD (Fig. 5*E*) as a function of guanidine hydrochloride. Although all CRPs have indistinguishable tryptophan emission and CD spectra in their native state, the unfolding titrations revealed important differences. Experiments monitoring changes in tryptophan fluorescence show that the CRP_BCG_ and CRP_MTB_–E178K have lower unfolding free energies (free energy change [ΔG°] ∼ 2.0 kcal mol^−1^) than the CRP_MTB_ and CRP_MTB_–L47P (ΔG° ∼ 2.6 kcal mol^−1^) (Table 5). It is possible that the lower stability is due to mutational perturbations of the tryptophan residue (W202) located in the DNA-binding domain, which is in close proximity to E178K. Experiments monitored with CD showed a different pattern: CRP_MTB_–L47P has the lowest unfolding free energy (ΔG° ∼ 3.9 kcal mol^−1^) and the smallest *m*-value (*m*) (–2.5 kcal mol^−1^ M^−1^) compared with the other proteins (Table 5). L47 is a fully buried residue (0% accessible surface area), and therefore, mutations in this position may contribute to a global destabilization of the protein. Interestingly, the unfolding curve of the CRP_BCG_ monitored by CD displayed two clear unfolding transitions (Fig. 5*E*). All the other CRPs displayed one unfolding transition, which was analyzed using a two-state unfolding model. These results again illustrate the nonlinear contributions and effects of the individual mutants to the CRP_BCG_. Given that unfolding experiments were conducted using 5 μM of protein, which is three times lower than its dimerization constant, it is possible that the two transitions observed for the CRP_BCG_ represent unfolding transitions of monomers and dimers in the solution.

**Table 5:** Thermodynamic stability of the CRP_MTB_, CRP_BCG_, and single mutants

CRP protein	CD			Fluorescence		
	ΔG°	*m*	C_1/2_	ΔG°	*m*	C_1/2_
MTB	5.4 ± 0.8	–3.4 ± 0.4	1.6 ± 0.4	2.6 ± 0.3	–1.9 ± 0.2	1.5 ± 0.4
L47P	3.9 ± 0.9	–2.5 ± 0.5	1.6 ± 0.7	2.6 ± 0.4	–1.7 ± 0.2	1.4 ± 0.3
E178K	6.3 ± 0.9	–3.8 ± 0.5	1.7 ± 0.5	1.8 ± 0.4	–1.5 ± 0.1	1.2 ± 0.4
BCG	ΔG_1_° 2.9 ± 0.5	*m*_1_: –4.2 ± 0.6	0.7 ± 0.2	2.0 ± 0.4	–1.6 ± 0.2	1.3 ± 0.4
	ΔG_2_° 3.4 ± 2.1	*m*_2_: –2.1 ± 1.1	1.6 ± 1.8			

BCG, Bacille Calmette-Guérin; CRP_BCG_, cAMP receptor protein from *Mycobacterium bovis* Bacille Calmette-Guérin strain; CRP_MTB_, CRP from *Mycobacterium tuberculosis*; ΔG°, free energy change; MTB, *Mycobacterium tuberculosis.*

The error corresponds to the SD from fitted parameters using a two-state and three-state models as described in Experimental procedures. The units of ΔG°, *m* (m-value), and C_1/2_ (unfolding transition midpoint) are kcal·mol^−1^, kcal mol^−1^ M^−1^, and M, respectively.

## Discussion

Elucidating the role of cAMP signaling in *M. tuberculosis* is a biomedically important topic because cAMP plays an important role in virulence and host interactions (4). Despite the relevant role that the CRP_MTB_ plays in cellular processes, there is limited information regarding its mechanism of allosteric regulation of transcription by cAMP. This is in contrast to the well-studied CRP_Ecoli_, which shares high sequence and structural similarity with the CRP_MTB_: ∼ 53% sequence similarity and an r.m.s.d = 2.5 Å between all atoms in the cAMP-bound structures (9, 11, 35). In this study, we use several biophysical approaches to investigate the linkage between cAMP binding and DNA interactions in the CRP_MTB_.

### Physiological role of cAMP and the CRP_MTB_ in *M. tuberculosis*

To survive the host's defense mechanisms, *M. tuberculosis* has developed a number of strategies that include the following: (1) interfering with phagosomal acidification and trafficking, (2) blocking autophagy and apoptosis-mediated killing, (3) perturbing calcium signaling, and (4) inhibiting inflammatory responses by modulating the host cytokine defenses and quenching the production of reactive oxygen and nitrogen species (5, 36). Some of these strategies can be accomplished by elevating levels of cAMP inside the host cell. Elevated levels of cAMP can suppress innate immune functions by modulating protein expression of inflammatory mediators, dampening the phagocytic response, and reducing intracellular killing of ingested pathogens (5). The best studied microbial strategy for elevating cAMP levels inside the host is by producing toxins that include adenylyl cyclases themselves (*M. tuberculosis* has 17, compared with *E. coli* that has 1). One such adenylyl cyclases is Rv0386, which is linked to the production and secretion of cAMP within macrophages and whose deletion decreases *M. tuberculosis* virulence and pathology in mice (37, 38). By using ^14^C-radiolabeled *M. tuberculosis*, Agarwal *et al*. showed that the increase in cAMP was mediated by the bacteria rather than by the host macrophages, and it was dependent on Rv0386 (37).

While the intracellular concentration of cAMP in *E. coli* has been well determined (1–40 μM) (39, 40, 41), reports on the concentration of the cyclic nucleotide in *M. tuberculosis* and *M. bovis* show variation between 0.5 and 7 pmoles per 10^8^ bacteria, depending on the growth media (42). *M. tuberculosis* has an irregular shape, ranging between a length of 2 and 4 μm and a width of 0.2 and 0.5 μm (43). Using this information and assuming a rod-shape morphology (43), the concentration of cAMP has lower and upper boundaries of 6.3 μM to 1.1 mM. Our cAMP- and DNA-binding studies indicate that at the lowest cAMP concentrations, CRP_MTB_–DNA oligomers formed *via* nonspecific interactions will be the dominant species, whereas at the highest concentration range, these oligomers will be lowly populated. Interestingly, the levels of cAMP inside *M. tuberculosis* from infected macrophages were reported to be 20 pmoles per 10^8^ bacteria, which by a similar calculation as above result in an intracellular cAMP concentration range between 0.26 and 3.2 mM (42). At those concentrations, the reduction of CRP_MTB_–DNA oligomers will be almost complete. Thus, the modulation of the cAMP concentration before and after macrophage infection will be accompanied by direct effects over the interactions between the CRP_MTB_ and DNA.

### cAMP is an allosteric modulator of DNA-binding specificity

We provide evidence for a previously unrealized role of cAMP signaling, in which cAMP regulates the specificity of CRP_MTB_–DNA interactions. This is in contrast to its structurally conserved CRP_Ecoli_ homolog, wherein cAMP controls the binding affinity to sequence-specific promoters (18, 19, 20, 44). This new role of cAMP in the CRP_MTB_ activation is supported by four experimental observations: first, fluorescence anisotropy experiments quantitatively show that the difference in CRP_MTB_–DNA affinities in the presence and absence of cAMP are marginal, a result that is in agreement with previous studies (7). This indicates that the bound cyclic nucleotide does not regulate transcription at the level of affinity to specific DNA promoter sequences (Fig. 3*A*, Table 3). Second, the observed anisotropy at high protein concentrations (relative to the concentration of DNA) is significantly higher in the absence of cAMP than in its presence. This difference is related to the formation of high-order CRP_MTB_–DNA oligomers that are prevented in the presence of cAMP or reversibly dissociated by adding cAMP after high-order CRP_MTB_–DNA oligomers are formed (Fig. 3, *C* and *D*). Third, from stoichiometric DNA-binding assays (Fig. 3*B*), we conclude that high-order CRP_MTB_–DNA oligomers only appear after 1-to-1 CRP_MTB_–DNA complexes have formed (*i.e.*, one CRP dimer and one DNA promoter). This result indicates that the absence of cAMP increases the affinity for nonspecific interactions between a preformed CRP_MTB_–DNA complex and apo-CRP_MTB_, between two preformed CRP_MTB_–DNA complexes, or both. Future AUC experiments will address the size distribution of these oligomers to determine their stoichiometries and relative populations. Fourth, we provide experimental evidence showing that a single cAMP molecule per CRP_MTB_ dimer prevents nonspecific DNA interactions and reverse the formation of high-order CRP_MTB_–DNA oligomeric complexes.

It is important to note that the aforementioned role of cAMP in regulating DNA-binding specificity occurs at concentrations of the CRP_MTB_ higher than 0.1 μM (Fig. 3*A* and Fig. 4, *B*, *C*, and *E*). If we consider the intracellular concentration of the CRP_MTB_ similar to that reported for the CRP_Ecoli_, 2.5 μM (40), then CRP_MTB_–DNA oligomers will readily form when the concentration of cAMP is in the low micromolar range (<50 μM). From previous reports (42), we estimated the concentration of cAMP ranging between 6.3 μM and 1.1 mM. CRP_MTB_–DNA oligomers will be present at the lowest levels of cAMP, while dissociating at the highest level of cAMP from the range provided. These protein and cAMP concentration estimates therefore underscore the biological relevance of cAMP in regulating the CRP_MTB_ specificity toward DNA sequences.

### The ability of cAMP to modulate DNA-binding specificity is lost in *M. bovis* BCG

The CRP ortholog of the attenuated *M. bovis* BCG (CRP_BCG_), whose sequence only differs in two amino acids at positions L47P and E178K relative to the CRP_MTB_, exhibits significant differences in gene regulation (29). Furthermore, previous studies have shown that the CRP_BCG_ has slightly higher DNA-binding affinities than the CRP_MTB_ for the same promoter sequences (29). Studies dissecting the role of each mutation site in the CRP_BCG_ showed that L47P, located at the cAMP-binding domain, had a greater effect in decreasing the ability of the protein to repress gene expression than E178K, located at the DNA-binding domain (30). Given these results, it has been proposed that the mutations observed in the CRP_BCG_ play a significant role in the attenuation of *M. bovis* BCG (30). Here we investigated the mechanisms by which the CRP_BCG_ differs from the CRP_MTB_ in its interactions with cAMP and DNA.

We find that the CRP_BCG_ binds the SerC promoter with a slightly higher affinity (∼2-fold) than the CRP_MTB_ (Table 4). However, the presence of cAMP for the CRP_BCG_ has no effects on the prevention of formation of high-order CRP–DNA oligomers (Fig. 4*C*). This result indicates that the allosteric control exerted by cAMP is largely reduced by the mutations L47P and E178K found in the CRP_BCG_. Which of these mutations is responsible for this new behavior? When the two mutations were investigated individually, we found that CRP_MTB_–L47P and CRP_MTB_–E178K behave similarly to the CRP_MTB_, namely, that the presence of cAMP reduced the formation of high-order CRP–DNA oligomers (Fig. 4, *E* and *F*). Thus, our results suggest that the functional characteristics of the CRP_BCG_ are not the consequence from a linear contribution of each individual mutant; rather, it is the result of cooperative interactions between the two mutation sites. For instance, the change in ANS emission due to cAMP binding is twice as large as compared with the single mutants. This is due to higher initial ANS–protein complex emission for the CRP_BCG_ (data not shown), indicating that ANS has a different mode of interaction or that more ANS molecules bind to the CRP_BCG_, or both. In agreement with this conclusion, the thermodynamic stability and dimerization *K*_d_ of the CRP_BCG_ are different from that of the CRP_MTB_ or the single mutants, indicating long-range interactions between the two mutation sites that give rise to unique functional and biophysical characteristics.

### Potential biological role for negative cAMP-binding cooperativity and DNA-binding specificity in transcription regulation mediated by the CRP_MTB_

What are the functional consequences of having negative cAMP-binding cooperativity and regulation of DNA-binding specificity in the CRP_MTB_? The observed negative cooperativity during cAMP-binding dictates that the probability of binding a second cAMP molecule to a singly cAMP-bound CRP_MTB_ dimer is lower than the probability of binding an apo-CRP_MTB_ dimer, resulting in a larger population of the singly cAMP-bound state. Given our results indicating that a single cAMP molecule bound to a CRP_MTB_ dimer reverses high-order CRP_MTB_–DNA oligomeric complexes, we hypothesize that negative cAMP-binding cooperativity may maximize the pool of available cAMP to favor sequence-specific interactions between the CRP_MTB_ and DNA. Thus, instead of requiring a doubly cAMP-bound CRP_MTB_ to reduce nonspecific interactions or reverse high-order CRP_MTB_–DNA oligomeric complexes, our results indicate that one cAMP molecule is sufficient to achieve the same outcome.

**Figure 6:**
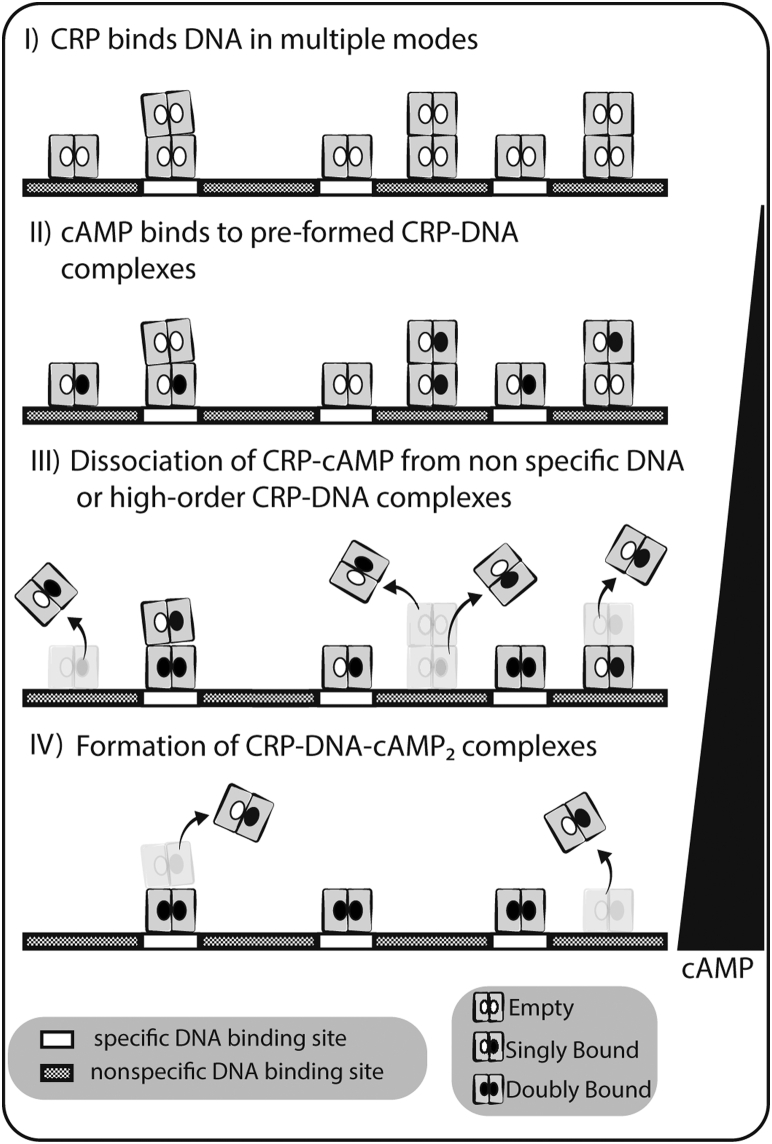
**Proposed cAMP allosteric signaling mechanism in the CRP**_**MTB**_. (I) the CRP_MTB_ binds to both specific and nonspecific DNA sequences in the absence of cAMP or forms high-order CRP_MTB_–DNA complexes. (II) cAMP binds to preformed CRP_MTB_–DNA complexes. (III) cAMP triggers the dissociation of the CRP_MTB_ from nonspecific DNA sites or dissociation of high-order CRP_MTB_–DNA oligomers into 1-to-1 complexes. Note that only one cAMP per CRP_MTB_ is sufficient to break nonspecific interactions. (IV) Saturated state of the CRP_MTB_ (two cAMP molecules bound) remains bound to specific DNA promoter sequences. CRP_MTB_, CRP from *Mycobacterium tuberculosis.*

The results from this study provide a previously unrecognized archetype of cAMP-mediated regulation of transcription that is different from previously described models for other CRPs. Figure 6 shows a model by which cAMP allosterically regulates CRP_MTB_ interactions with DNA: (I) In the absence of cAMP, the CRP_MTB_ binds to both specific (*i.e.*, promoters) and nonspecific (*i.e.*, intragenic) sequences of DNA or forms high-order CRP_MTB_–DNA complexes. (II) When cAMP levels increase inside the cell after macrophage infection, cAMP binds to a preformed CRP_MTB_–DNA complex. Because of the negative cooperativity of the CRP_MTB_, the first cAMP-binding event dominates over the second; thus, cAMP is preferably bound to a single subunit within homodimers. (III) While singly cAMP-bound proteins dissociate from nonspecific DNA sequences or dissociate high-order CRP_MTB_–DNA oligomers into 1-to-1 complexes, proteins that were interacting with specific DNA sequences remain bound to its promoter. (IV) Finally, as the cAMP concentration increases, the doubly cAMP-bound state is reached with mostly specific CRP_MTB_–DNA interactions taking place.

This model offers three scenarios by which the CRP_MTB_ may regulate transcription and underscores its role as a global regulator. First, like removing roadblocks along the DNA structure (45, 46), cAMP will trigger the dissociation of the CRP_MTB_ from nonspecific DNA sequences. Second, CRP_MTB_–cAMP will remain bound to specific promoter sequences, facilitating the recruitment of the transcription machinery such as other transcription factors or RNA polymerase. Third, and a less studied role attributed to the CRP_MTB_, is chromosome organization (26). The CRP_MTB_ binds to >900 sites in the *M. tuberculosis* genome, 83% of which are intragenic regions (47). This type of binding resembles that of nucleoid-associated proteins and suggests that the CRP_MTB_ might regulate the global architecture of the mycobacterial chromosome (48). The ability of the CRP_MTB_ to bend DNA (6) could also alter the interaction mode of other factors that interact to DNA proximal to CRP sites. In the context of our results, the property of CRP_MTB_–DNA to form oligomeric complexes and then dissociate as a function of cAMP concentration might be a strategy for regulating gene expression *via* chromosomal organization. Altogether, the interplay between these three mechanisms results in the expression of genes involved in virulence, such as ESX-1 type VII secretion system (T7SS), espACD-Rv3613c-Rv3612c operon, Rv3616c-Rv3612c genes, espA operon, to name a few, all of which are associated to CRP_MTB_ activity (26). The CRP_MTB_ also activates expression of *rpfA* and *whiB1* genes that encode proteins that are thought to be involved in reviving dormant bacteria (12, 25, 26, 49). Within this model, our results indicate that the variant CRP_BCG_ has difficulties dissociating from nonspecific DNA sequences or reverse the formation of high-order CRP–DNA oligomers, possibly obstructing transcription.

### Similar structures, different allosteric activation mechanisms

It is intriguing that the CRP_MTB_ and CRP_Ecoli_ share high sequence and structural similarity (11, 35) but differ in their cAMP-mediated activation mechanisms. Although high-resolution structures indicate that these two homologs are cAMP-dependent transcription factors, it is more difficult to infer from the structures alone that these proteins would have very different cAMP-binding modes and cAMP-dependent DNA interactions. A close inspection of the two CRP structures reveals small differences that may be associated with their unique allosteric properties. For instance, the carboxy-terminal residues of the CRP dimerization helix in the apo-CRP_Ecoli_ are not well structured, whereas in the CRP_MTB_ they are (Fig. 1*A*). These residues are part of the hinge that connects the cAMP- and DNA-binding domains and have been shown to contribute to the allosteric communication in the CRP_Ecoli_ (50, 51, 52). Moreover, cAMP-induced domain motions in the CRP_MTB_ originate at the hinge that connects the cAMP-binding domain and the dimerization helix. Instead, in the CRP_Ecoli_ domain, motions originate at the hinge that connects the DNA-binding domain and the dimerization helix (Fig. 1, *B* and *D*). The interplay between the sequence composition and the location of these domain motions may help further dissect the unique allosteric behavior in the CRP_MTB_ and how cAMP reduces nonspecific DNA interactions.

Given that the structures of the CRP_MTB_ in the apo-state and cAMP-bound state are similar, it is plausible that protein dynamics (16, 18, 21, 53, 54, 55, 56) also play an important role in how cAMP allosterically reduces nonspecific DNA interaction. Our cAMP-binding studies show differences in ANS fluorescence between apo-state and cAMP-bound state (Fig. 4*A*), despite their similar structures (Fig. 1, *A* and *B*, *bottom*). This change in ANS fluorescence indicates dynamic transitions or protein fluctuations associated with cAMP binding that are not captured in static high-resolution structures. Future studies with high-resolution techniques such as hydrogen–deuterium exchange mass spectrometry (57, 58) will help elucidating the residue networks involved in cAMP-mediated allostery and communication.

It is well documented that the CRP_Ecoli_ exhibits positive cooperativity between the two cAMP-binding sites, wherein the first binding reaction is exothermic and the second is endothermic (18, 19, 24, 33). In contrast, by using two orthogonal techniques (ITC and fluorescence), we find that the CRP_MTB_ displays negative cAMP-binding cooperativity (Fig. 2, Table 1), where binding of the cyclic nucleotide is endothermic for both sites.

A previous study with ITC by Stapleton *et al*. (12) reported that the two cAMP-binding events in the CRP_MTB_ are independent from each other. Our cAMP-binding studies using ITC and ANS fluorescence and the underlying statistical analysis of the data (Table S2) do not agree with their results. A potential source for the difference is that we used a cAMP-to-CRP_MTB_ molar ratio of up to 5-fold for ITC experiments, whereas Stapleton *et al*. reached a maximum ratio of 2.5. Given the expected 2-to-1 binding stoichiometry between cAMP and the CRP_MTB_, we used a higher molar ratio to ensure full saturation of the protein at the end of the titration. Alternatively, the buffer type and composition could be a source of differences between the two studies. We conducted ITC experiments at a constant pH using three buffers with different ionization enthalpies, including PBS, which was used by Stapleton et al. In all three experiments, we maintained a cAMP-to-CRP_MTB_ molar ratio up to 5-to-1 and obtained the same degree of negative cooperativity between the two cAMP-binding sites (Table 2, Figs. S1 and S2, and Tables S1 and S2). Altogether, the three ITC experiments and fluorescence measurements used in this study are consistent with each other and strongly suggest that cAMP binds with negative cooperativity to the CRP_MTB_.

### Concluding remarks

**Figure 7:**
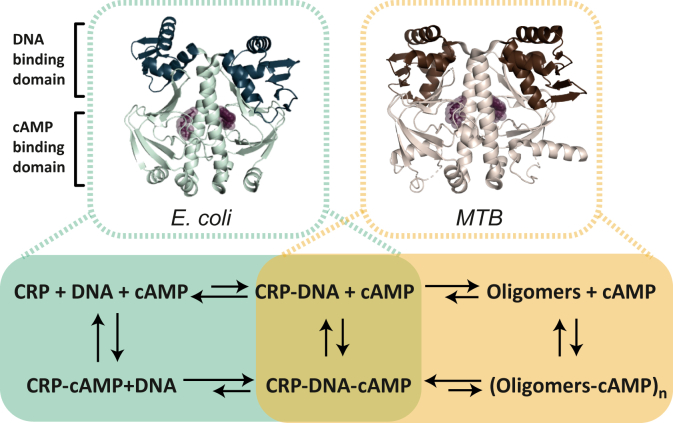
**Comparison between the cAMP-induced allosteric effects in the CRP**_**Ecoli**_**and CRP**_**MTB**_. The *top panel* shows the crystal structures of the CRP_Ecoli_ and CRP_MTB_ bound to cAMP (*purple spheres*). The *lower* panel shows the thermodynamic cycles that underlie the allosteric effect of cAMP in both proteins. For the CRP_Ecoli_, DNA-binding events are more favorable (*i.e.*, have higher affinity) in the presence of cAMP. In contrast, CRP_MTB_–DNA complexes are formed with similar affinity with and without cAMP. Nonspecific DNA interactions and high-order CRP_MTB_–DNA oligomers are either reduced or prevented by cAMP, promoting the formation of a stable one-to-one CRP_MTB_–DNA complex. CRP_Ecoli_, CRP from *Escherichia coli*; CRP_MTB_, CRP from *Mycobacterium tuberculosis.*

In this study, we begin to dissect the linkage between cAMP binding and DNA interactions in the CRP_MTB_. Importantly, this study indicates that the linkage operates at a level of DNA regulation that is substantially different to that from the CRP_Ecoli_. Figure 7 illustrates the functional differences between the CRP_MTB_ and CRP_Ecoli_ at the level of DNA interactions. Although cAMP enhances the affinity of the CRP_Ecoli_ for DNA promoter sequences and promote intersubunit communication (10, 13, 19, 23, 24, 59, 60), our results from this study show that DNA-binding affinity to the CRP_MTB_ is not sensitive to cAMP occupancy. Instead, cAMP plays a significant role on the specificity of DNA interactions and the reduction or prevention of high-order CRP-DNA oligomers. The exact functional consequences of such a mode of action will likely depend on the specific organization of regulatory elements for a particular gene (12) and the degree of energetic coupling between the four binding sites in CRP_MTB_, *i.e.*, binding at any site has the potential to alter the binding affinity of the other three sites.

## Experimental procedures

### R.M.S.D. analysis of structures of the CRP_Ecoli_ and CRP_MTB_

Structural analyses were performed using PyMol Molecular Graphics System (Version 2.0 Schrödinger, LLC.). The Protein Data Bank used here were 2WC2 and 1G6N for CRP_Ecoli_ in the apo-state and cAMP-bound state, and 3D0S and 3I54 for CRP_MTB_ in the apo-state and cAMP-bound states. The cAMP-binding domain alignment (residues 21–104 in CRP_Ecoli_ and residues 28–110 in CRP_MTB_) between intraspecies subunits or between interspecies subunits served as an anchor for all the r.m.s.d. values reported in this study.

### Cloning, expression, and purification of the CRP_MTB_

The DNA sequence of WT CRP from *M. tuberculosis* (CRP_MTB_) was used in the present study. CRP flanked by NdeI and BamHI restriction sites was synthesized by PCR with *Pfu*Ultra Polymerase (Agilent Technologies). The amplicon was digested with NdeI and BamHI according to manufactured directions (New England Biolabs). To generate the His-tag fusion construct, the resultant digested fragment was inserted into a pET-3a expression vector (Addgene) previously digested with the same restriction enzymes. The resultant expression vector was named the CRP_MTB_.

CRP_MTB_ mutants (E178K, L47P, and BCG [E178K/L47P double mutant]) were generated following the QuikChange II Site-Directed Mutagenesis protocol (Agilent Technologies). All proteins were purified from *E. coli* strain T7 Express pLysS competent cells (New England Biolabs). The bacteria were grown overnight, and protein expression was induced with 1-mM IPTG for 2 h. The bacteria were resuspended in an ice-cold lysis buffer (20-mM Tris, 200-mM NaCl; 10 ml/g of wet weight) supplemented with protease inhibitors (10-mM benzamidine, 0.4-mM AEBSF, 1-μM pepstatin, 1-μM leupeptin, 28-μM Tosyl phenylalanyl chloromethyl ketone and and Tosyl-L-lysine chloromethyl ketone, 10-μM 3-isobutyl-1-methylxanthine, 1-mM PMSF). The bacterial suspension was homogenized with a glass homogenizer and lysed with an M-110P Microfluidizer at 10,000 psi (Microfluidics). The lysate was centrifuged at 15,000 rpm for 45 min at 4 °C in a JA 25.50 rotor (Beckman Coulter). The supernatant was mixed with the His60 Nickel Superflow Resin (Clontech) and allowed to bind overnight at 4 °C with constant shaking. The supernatant was supplemented with 30-mM imidazole to compete for nonspecific binding. The next day, the resin–supernatant mix was transferred to a prewashed column with the lysis buffer. The flow-through sample was collected, and the resin was washed twice with lysis buffer supplemented with 3-mM imidazole. 500-mM imidazole was added in the lysis buffer, the resin was incubated for 30 min, and the elutes were collected. Samples corresponding to the CRP_MTB_ were pooled together, concentrated, and run through size-exclusion chromatography. All proteins in the apo-state had elution profiles displaying a single peak at elution volumes consistent with a dimeric state (Fig. S8). Aliquots were taken at every step of the purification protocol and loaded on to 10% SDS-PAGE gels to follow the purification process and assess the quality of the purified protein. The purified proteins were >95% homogenous as judged by SDS-PAGE. The CRP_MTB_ were stored at −80 ^o^C in the storage buffer (50-mM Hepes, pH 7.6, 150-mM KCl, 1-mM EDTA, pH 7.2). Protein concentrations throughout this study were determined with the dimer extinction coefficient at 280 nm: 25,480 cm^−1^ M^−1^.

### CD

Measurements were performed in an Aviv Model 202-01 spectrometer with 5-μM protein in a buffer containing 150-mM KCl, 50-mM Hepes, and 1-mM EDTA, pH 7.2, over a range of 195 to 260 nm. For each sample, three scans with three different protein samples preparations were performed, averaged, and baseline-corrected.

### Chemical denaturation with guanidine hydrochloride

Protein unfolding was monitored by changes in fluorescence (λ_ex_ = 280 nm or 295 nm, and λ_em_ = 340 nm) and CD absorption at 222 nm. In both set of experiments, we used 5 μM of protein in a buffer containing 150-mM KCl, 50-mM Hepes, and 1-mM EDTA, pH 7.2. At least two independent titrations were performed for each protein and corrected for buffer contributions to the signal. Data were fitted according to the linear extrapolation method (61). For the WT CRP_MTB_ and single mutants CRP_MTB_–E178K and CRP_MTB_–L47P, the data were fitted to a two-state unfolding model (61):

(1) $$S_{T}=S_{N}f_{N}+S_{D}f_{D}$$

where *S*_*T*_ is the total observed signal, *S*_*N*_ and *S*_*D*_ correspond to the native and denatured state signals, respectively, and *f*_*N*_ and *f*_*D*_ are the fractions of native and denatured protein, respectively. *f*_*N*_ and *f*_*D*_ are related to the equilibrium constant between folded and unfolded states:

(2) $$f_{N}=\frac{1}{1\;+\;K}$$

(3) $$f_{D}=\frac{K}{1\;+\;K}$$

where:

(4) K=e−ΔGoRT

And:

(5) $$\Delta G^{o}=\Delta G_{H_{2}O}^{o}+m\left[d\right]$$

Here, $\Delta G_{H_{2}O}^{o}$ is the free energy of unfolding in the absence of a denaturant, *m* is the m-value or the slope of the linear dependence of $\Delta G^{o}$ on the denaturant concentration as described by the linear extrapolation method (61), and *d* is the denaturant concentration. Combining Equations (1), (2), (3), (4), (5) yields the data fitting equation:

(6) $$S_{T}=\frac{S_{N}\;+\;S_{D}e^{-\left(\frac{\Delta G_{H_{2}O}^{o}\;+\;m\left[d\right]}{RT}\right)}}{1\;+\;e^{-\left(\frac{\Delta G_{H_{2}O}^{o}\;+\;m\left[d\right]}{RT}\right)}}$$

The unfolding data for the CRP_BCG_ displayed two transitions, and therefore, the total signal, *S*_*T*_, was fitted to a three-state unfolding model (62):

(7) $$S_{T}=S_{N}f_{N}+S_{I}f_{I}+S_{D}f_{D}$$

where *S* and *f* are the signals and fractions of native (*N*), intermediate (*I*), and denatured (*D*) states, respectively. Expressing the fractions of species in terms of equilibrium constants yields:

(8) $$f_{N}=\frac{1}{1\;+\;K_{I}\;+\;K_{I}K_{D}}$$

(9) $$f_{I}=\frac{K_{I}}{1\;+\;K_{I}\;+\;K_{I}K_{D}}$$

(10) $$f_{D}=\frac{K_{I}K_{D}}{1\;+\;K_{I}\;+\;K_{I}K_{D}}$$

where *K*_I_ and *K*_D_ are the equilibrium constants between the native and intermediate states and the intermediate and denatured states, respectively. *K*_I_ and *K*_D_ are expressed in terms of $\Delta G_{H_{2}O}^{o}$ and *m* as in Equation 5 for intermediate (subscript *I*) and denatured (subscript *D*) states, resulting in the following equation:

(11) $$S_{T}=\frac{S_{N}\;+\;S_{I}e^{-\left(\frac{\Delta G_{H_{2}O,\;I}^{o}\;+\;m_{I}\left[d\right]}{RT}\right)}\;+\;S_{D}e^{-\left(\frac{\Delta G_{H_{2}O,\;I}^{o}\;+\;m_{I}\left[d\right]}{RT}\right)}e^{-\left(\frac{\Delta G_{H_{2}O,D}^{o}\;+\;m_{D}\left[d\right]}{RT}\right)}}{1\;+\;e^{-\left(\frac{\Delta G_{H_{2}O,\;I}^{o}\;+\;m_{I}\left[d\right]}{RT}\right)}\;+\;e^{-\left(\frac{\Delta G_{H_{2}O,\;I}^{o}\;+\;m_{I}\left[d\right]}{RT}\right)}e^{-\left(\frac{\Delta G_{H_{2}O,D}^{o}\;+\;m_{D}\left[d\right]}{RT}\right)}}$$

To better compare the unfolding data of the four CRPs studied here, we plotted the fraction of folded protein. All fitting procedures of unfolding data were performed in SigmaPlot (Systat Software).

### Analytical ultracentrifugation

SV experiments were performed in a Beckman Optima XLA with absorbance optics in 12-mm cells at 280 nm, 50,000 rpm, and 19.7 ^o^C. The buffer density was measured in an Anton Paar DMA 5000. Extinction coefficients at 280 nm (12,740 M^−1^ per monomer or 0.4855 ml/mg) and vbar (0.735639) are estimated from amino acid sequence in Sednterp (63). Three samples were run at approximately 0.2, 0.4, and 0.6 absorbance at 280 nm (equivalent to dimer CRP concentrations of 7.9, 15.8, and 23.7 μM) in 150-mM KCl, 50-mM Hepes, and 1-mM EDTA, pH 7.2. Data were analyzed with DCDT+ (64) to generate g(s) distributions and plotted *versus* s∗ (Fig. 5*C*). Superposition of the WT CRP_MTB_ and single mutants CRP_MTB_–E178K and CRP_MTB_–L47P g(s) curves is consistent with no concentration dependence in the concentration regime tested. Data were then fit with SedAnal (version 7.14) (65) to determine global S values and for the CRP_BCG_ dimerization constants. The WT CRP_MTB_ and single mutants CRP_MTB_–E178K and CRP_MTB_–L47P have an average S_20,w_ of 3.587 s ± 0.082 or 2.3% consistent with an estimate using HullRad (66), 3.65 s. The SV data for the CRP_BCG_ were fit to a monomer–dimer model constraining the dimer S2 value to the individual and average values for the dimeric constructs or float S1 values which constrain the ratio of S2/S1 to 1.5. The best value for the CRP_BCG_ dimerization is 5.7·10^4^ M^−1^ or a *K*_*d*_ of 17.5 μM (binding free energy -6.47 ± 0.73 kcal mol^−1^).

### cAMP binding monitored by ITC

Experiments were performed in a Nano-ITC (TA instruments) using three different buffers (pH 7.2): Hepes, PBS, and cacodylate. Each buffer was supplemented with 150-mM NaCl, 1-mM EDTA, and 0.2-mM TCEP. All solutions were filtered and degassed thoroughly prior use. The protein and cAMP concentrations were 16 to 20 μM and 1 mM, respectively. The cAMP solution was prepared in the buffer from the last step of protein dialysis to minimize artifacts due to differences in the buffer composition. The reaction cell contained 0.35 ml of the protein solution. The injection syringe was filled with cAMP, and the titration experiment consisted of 18 injections. The first injection was of 0.5 μl and was discarded from the analysis step. The other 17 injections were of 2 μl. A separate reference titration of the cAMP into each buffer was performed to determine the heat of dilution of the ligand which was then subtracted from the cAMP titration to the protein solution. Raw data were analyzed using the software NITPIC (67) and MicroCal Origin using two different models: independent and sequential cAMP-binding events. The incremental heat (Q_i_) of the titration was fitted using Equation 12:

(12) $$\Delta Q_{i}=Q_{i}+\frac{dV_{i}}{V_{0}}\left[\frac{Q_{i}\;+\;Q_{\left(i-1\right)}}{2}\right]-Q_{\left(i-1\right)}$$

where V_i_ and V_0_ are the initial and active volumes, respectively. For the independent binding model, the total heat (Q) is the following:

(13) $$Q=\frac{n{\left[M\right]}_{t}\Delta HV_{0}}{2}\left[1+\frac{{\left[x\right]}_{t}}{n{\left[M\right]}_{t}}+\frac{1}{nK{\left[M\right]}_{t}}-\sqrt{{\left(1+\frac{{\left[x\right]}_{t}}{n{\left[M\right]}_{t}}+\frac{1}{nK{\left[M\right]}_{t}}\right)}^{2}-\frac{4{\left[x\right]}_{t}}{n{\left[M\right]}_{t}}}\right]$$

where *n* is the number of binding sites, [*M*]_*t*_ is the bulk protein concentration, *ΔH* is the ligand-binding enthalpy, [*x*]_*t*_ is the total ligand concentration, and *K* represents the binding constant.

The total heat for the sequential binding model is calculated *via* Equation 14.

(14) $$Q={\left[M\right]}_{t}V_{0}\left[\frac{K_{1}\left[x\right]\Delta H_{1}\;+\;K_{1}K_{2}{\left[x\right]}^{2}\left(\Delta H_{1}\;+\;\Delta H_{2}\right)}{1\;+\;K_{1}\left[x\right]\;+\;K_{1}K_{2}{\left[x\right]}^{2}}\right]$$

where [*x*] is

(15) $$\left[x\right]={\left[x\right]}_{t}-{\left[M\right]}_{t}\left(\frac{K_{1}\left[x\right]\;+\;2K_{1}K_{2}{\left[x\right]}^{2}}{1\;+\;K_{1}\left[x\right]\;+\;K_{1}K_{2}{\left[x\right]}^{2}}\right)$$

Here, *K*_*1*_ and *K*_*2*_ correspond to the microscopic binding constants 2⋅*k*_1_ and 1/2⋅*k*_2_, respectively.

We performed one- and two-way ANOVA tests to determine differences between *ΔH* and the buffer used in ITC experiments. To compare pairwise differences between buffers, we used the post hoc Tukey test with a significance level of 0.05. *p*-Values resulting from these tests are indicated in the legend of Figure 2. These tests were performed in *Mathematica* (Wolfram Research, Inc).

### EMSA

Reaction mixtures contained 40 nM of six different lengths of the SerC promoter fragments (18, 20, 22, 24, 26, and 32 bps) and between 0.1- and 3.0-μM CRP_MTB_ in 75-mM KCl, 50-mM Hepes, and 1-mM EDTA at pH 7.6. Sequences for the SerC promoter fragments were as follows: 32-bp (5′-GCGCGTAGTGTGAACAAGCTCACATGCAAGCC-3′), 26-bp (5′-CGTAGTGTGAACAAGCTCACATGCAA-3′), 24-bp (5′-GTAGTGTGAACAAGCTCAC-ATGCA-3′), 22-bp (5′-TAGTGTGAACAAGCTCACATGC-3′), 20-bp (5′-AGTGTGAAC-AAGCTCACATG-3′), and 18-bp (5′-GTGTGAACAAGCTCACAT-3′). Underlined regions correspond to the CRP_MTB_-binding site in the SerC promoter fragment (29). After 45 min of equilibration at room temperature, the reaction mixtures were loaded in an 8.5% polyacrylamide gel with 0.5× Tris-borate-EDTA buffer. Gels were run at 80 V for 70 min in 0.5× Tris-borate-EDTA buffer.

### cAMP binding monitored by ANS fluorescence

cAMP binding to the CRP_MTB_ was measured by the quenching of the fluorescent signal from the CRP–ANS complex (λ_ex_ = 350 and λ_em_ = 480 nm), using a PTI spectrometer (Horiba). Normalized intensity counts as a function of the cAMP concentration were fitted to a cooperative two-site binding model as described (19) and an independent two-site binding model. The cooperative model is shown in Equation 16.

(16) $$F_{480\;nm}=\frac{F_{0}\;+\;2F_{1}k_{1}\left[x\right]\;+\;F_{2}k_{1}k_{2}{\left[x\right]}^{2}}{1\;+\;2k_{1}\left[x\right]\;+\;k_{1}k_{2}{\left[x\right]}^{2}}$$

where *F*_*480 nm*_ is the observed signal; *F*_*0*_, *F*_*1*_, and *F*_*2*_ represent the fluorescent signal of the apo, singly liganded, and doubly liganded states of the protein, respectively; *k*_*1*_ and *k*_*2*_ corresponds to the microscopic binding affinity constants of the first and second cAMP, respectively, and *x* is the concentration of ligand. In the independent binding model, *k*_*2*_ = *k*_*1*_ which assumes that the ligand binding sites are identical (*i.e.*, no cooperativity). The ANS-based fluorescence data is normalized to the initial fluorescence value in the absence of cAMP. DNA biding curves were fitted with Sigma Plot (Systat Software).

### Statistical analysis of cAMP binding data

We used the *f-ratio* function to determine which cAMP binding model is statistically more robust in fitting the data from ITC and ANS experiments. First, we determine *f*_*obs*_, which is the *f-ratio* calculated from the fitted parameters of cAMP binding data:

(17) fobs=(SSR1ν1)(SSR2ν2)

where *SSR* is the sum of square of the residuals and ν correspond to degrees of freedom. The subindexes 1 and 2 refer to model 1, which in this case is identical and independent binding sites with no cooperativity, and model 2, which corresponds to identical binding sites with cooperative interactions. The values for *SSR*, ν and *f*_*obs*_ are listed in Table S2. In its application, *f*_*obs*_ is compared to the cumulative distribution of the *f-ratio* function using the corresponding degrees of freedom for models 1 and 2. This comparison provides a means to reject the hypothesis that model 1 is statistically equivalent to model 2 with a given confidence interval (68). Additionally, we calculated the probability that a value selected randomly from the *f-ratio* probability distribution exceeds *f*_*obs*_, *i.e.*, the probability that model 1 provides a better fit than model 2. This is achieved by integrating the cumulative distribution of the *f-ratio* function from *f*_*obs*_ to infinity. The results are listed in Table S2. These tests were done in *Mathematica* (Wolfram Research, Inc).

### DNA binding monitored by fluorescence anisotropy

Measurements were collected with a PTI spectrometer using a 32-bp SerC promoter (5′-GCGCGTAGTGTGAACAAGCTCACATGCA-AGCC-3′), 20-bp SerC promoter (5′-AGTGTGAACAAGCTCACATG-3′) and Scramble 32-bp DNA (5′-AGATCCGCAACATGTGTCGAACACACGCGGTA-3′) covalently linked to a fluorescein molecule (IDTDNA). The excitation and emission wavelengths were 480 nm and 518 nm, respectively. The reaction mixture contained either 3 nM, 200 nM or 400 nM of fluorescein-labeled DNA and various concentrations of cAMP (0, 100 and 1000 μM). Fluorescence anisotropy measurements were collected with a PTI spectrometer (Horiba Scientific). Data was normalized to the first experimental anisotropy value, and analyzed as described previously by Heyduk and Lee (20) and Lanfranco *et al*. (19) with minor modifications. Briefly, we removed experimental data points displaying anisotropy values with 2 standard deviation higher that the plateau overserved after the first DNA-binding phase (indicated by the *red arrow* in Fig. 3*A*). The data were fitted according to Equation 18,

(18) $$A_{obs}=A_{DNA_{F}}+\left(A_{P-DNA}-A_{DNA_{F}}\right)\cdot \frac{K\left[DNA_{T}\right]+K\left[P_{T}\right]+1-\sqrt{{\left(K\left[DNA_{T}\right]+K\left[P_{T}\right]+1\right)}^{2}+4K^{2}\left[DNA_{T}\right]\left[P_{T}\right]}}{2K\left[DNA_{T}\right]}$$

where *A*_*obs*_ is the observed anisotropy, *A*_*DNAF*_ and *A*_*P-DNA*_ are the anisotropy values for free DNA and the protein–DNA complex, respectively, [DNA_T_] is the total DNA concentration, [P_T_] is the total protein concentration, and *K* represents the association constant for the protein and DNA.

## Data availability

Data not contained in this article are available upon request to Rodrigo Maillard (rodrigo.maillard@georgetown.edu, Georgetown University).

## Supporting information

This article contains supporting information.

## Conflict of interest

The authors declare that they have no conflicts of interest with the contents of this article.
