# Supplementary material for: cAMP is an allosteric modulator of DNA-binding specificity in the cAMP receptor protein from Mycobacterium tuberculosis
Source: J Biol Chem. 2021 Feb 26;296:100480. doi: 10.1016/j.jbc.2021.100480 (PMC8026907; doi:10.1016/j.jbc.2021.100480)
Supplement: Figures S1 to S8 — and Table S1 and S2 [file mmc1.pdf]

Supporting Figure S1

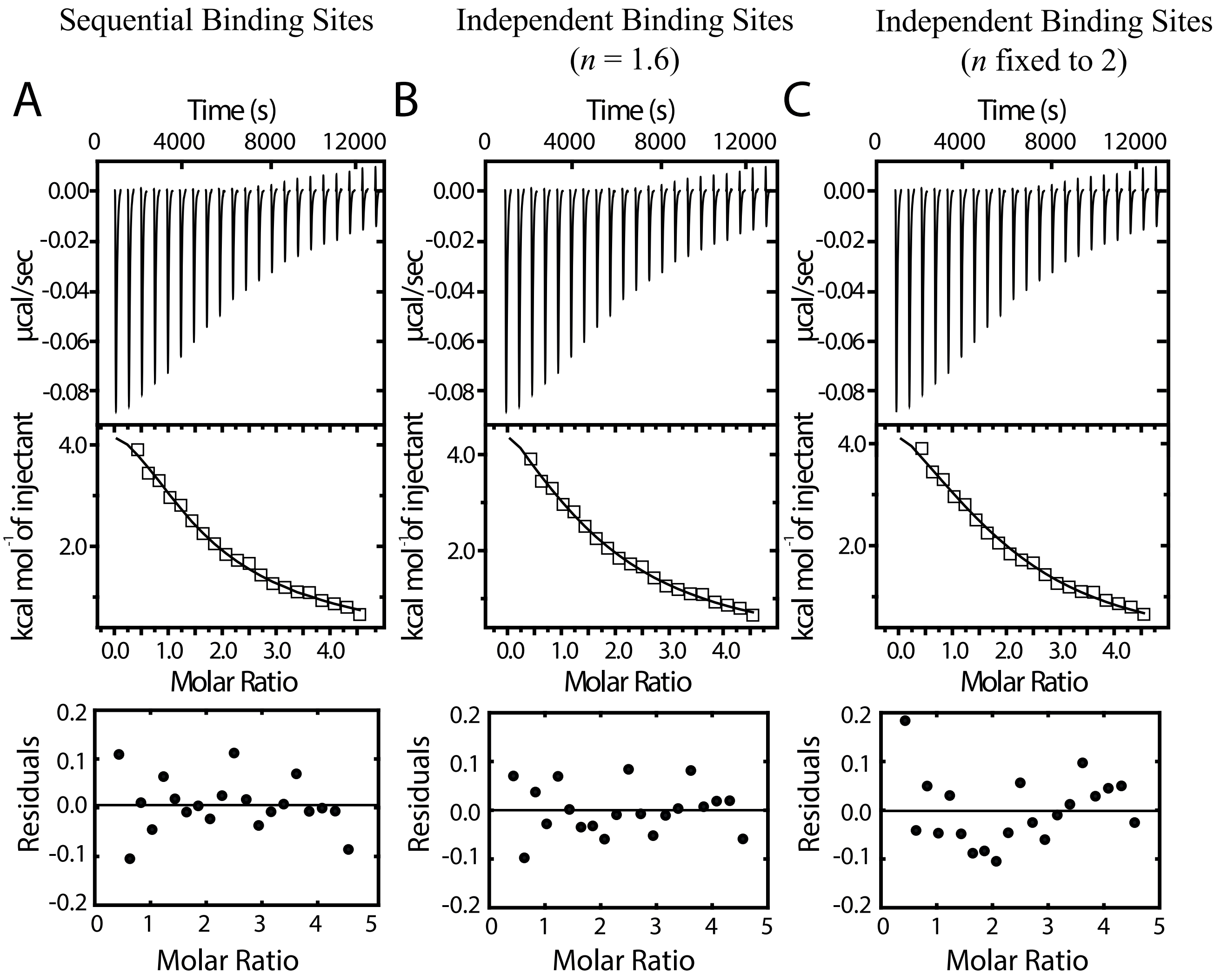

Supporting Figure S1. ITC data corresponding to sequential (A) and independent binding sites (B-C) models for $\mathbf{CRP_{MTB}}$ in HEPES buffer. Left panels corresponds to a two-site sequential binding model fitting. Middle panels corresponds to the independent sites fitting model where the resulting stoichiometry, *n*, was 1.6. Right panels uses the same model but with *n* fixed to 2, the expected stoichiometry for a homodimer with one cAMP-binding domain per subunit. Residuals of the fit were improved for a sequential model compared to an independent binding model with $n = 2$ (left vs. right panels). Residuals and fitting statistics for an independent model improved only when $n = 1.6$, which is a value that has no physical meaning for a homodimeric protein. The thermodynamic data, binding constants and fitting statistics are listed in **Table 1**, **Table 2**, and **Supporting Tables S1** and **S2**. The equations used for the different binding model mechanisms are described in Materials and Methods.

Supporting Figure S2

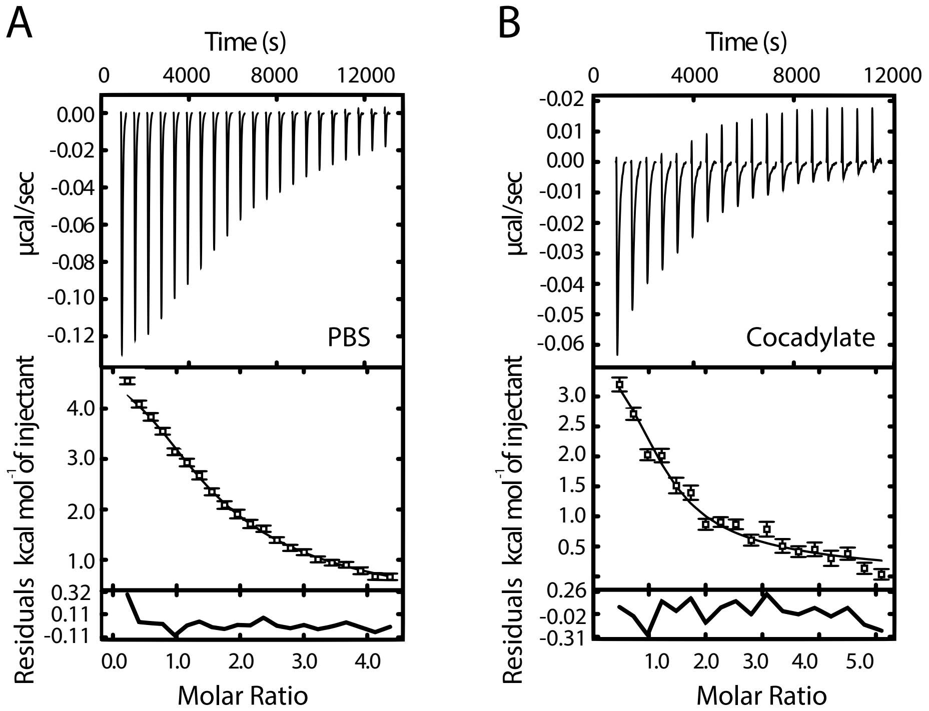

Supporting Figure S2. ITC data corresponding to sequential binding models in PBS (A) and Cacodylate (B) buffers. The thermodynamic data, binding constants and fitting statistics are listed in **Table 1**, **Table 2**, and **Supporting Tables S1** and **S2**. The equations used for the binding model mechanisms are described in Materials and Methods.

Supporting Figure S3

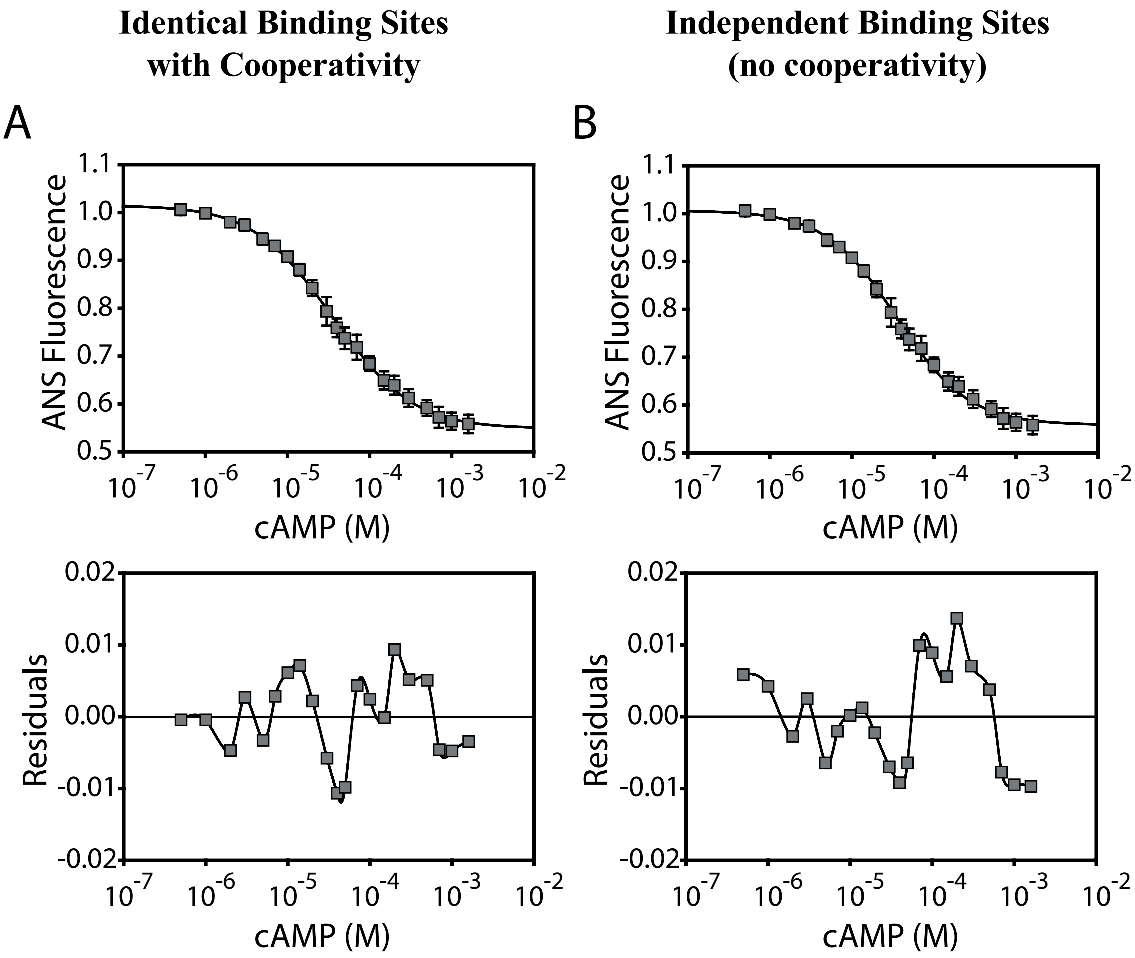

Supporting Figure S3. ANS fluorescence data for CRP WT fitted to a cooperative binding model (A) and independent binding model (B) with residuals. The light grey squares are normalized ANS fluorescence data for wild type CRP_{MTB}. The solid lines correspond to the fitted models. A model with identical binding sites with cooperativity resulted in microscopic binding constants, $k_1 = (3.3 \pm 0.2)\cdot 10^4\ M^{-1}$ and $k_2 = (2.2 \pm 0.1)\cdot 10^4\ M^{-1}$, which yields the cooperativity value $c = 0.6 \pm 0.1$. The independent model reported the binding constant $k = (2.80 \pm 0.01)\cdot 10^4\ M^{-1}$. Analysis using the *f-ratio* distribution indicates that a model with identical binding sites with cooperativity provides an statistically better fit (**Supporting Table S2**).

Supporting Figure S4

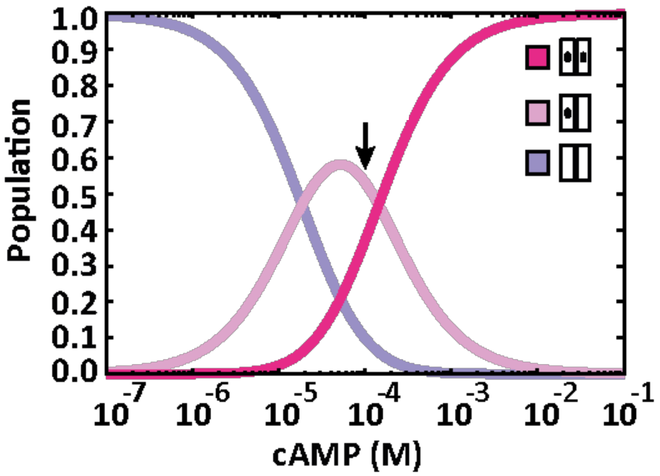

Supporting Figure S4. Simulation of the distribution of populations for CRP_{MTB} as a function of cAMP concentration. The light purple, light pink and pink lines correspond to the apo, singly and doubly cAMP-bound populations, respectively, simulated using the microscopic binding constants listed in **Table 1**. Arrow indicates the cAMP concentration 100 μM

Supporting Figure S5

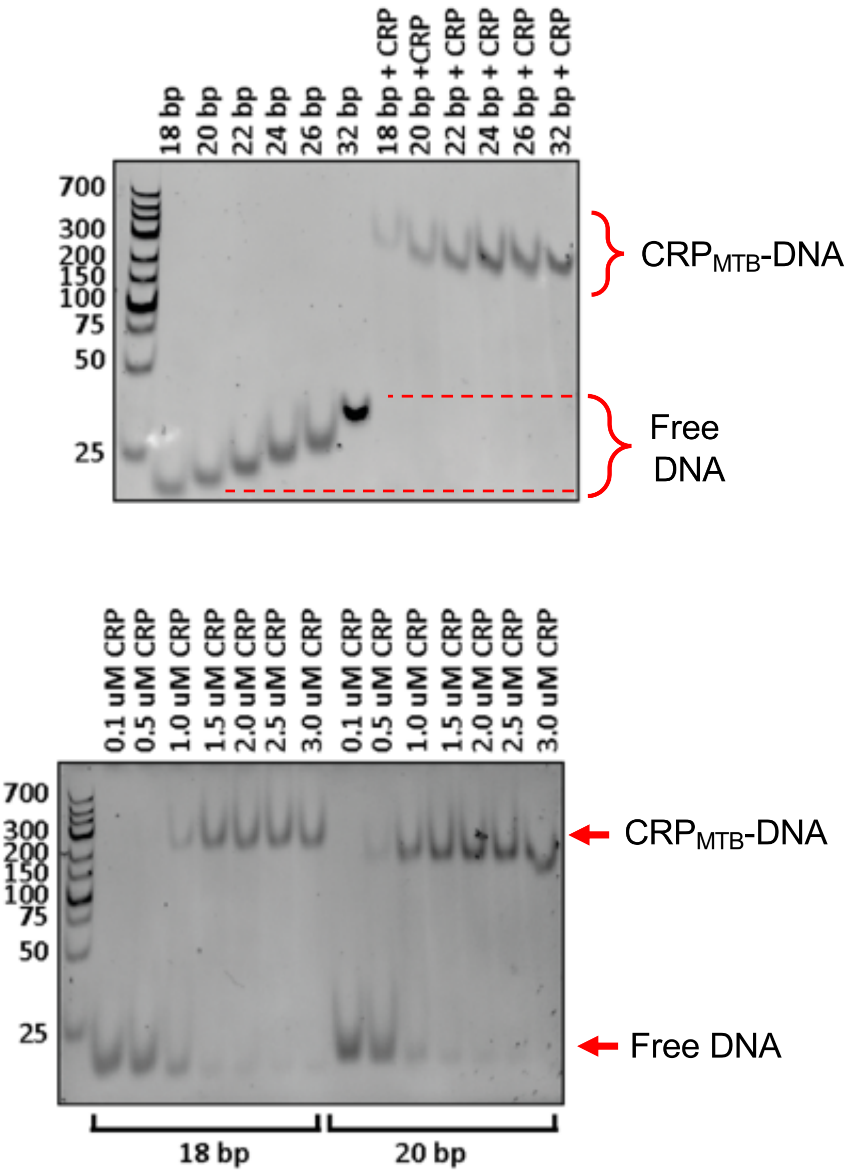

Supporting Figure S5. Binding of CRP_{MTB} to different SerC promoter lengths. Top panel shows the formations of CRP_{MTB}-DNA complexes using the SerC promoter of increasing lengths: 18, 20, 22, 24, 26 and 32-bp. Bottom panel shows the formation of CRP_{MTB}-DNA complex as a function of CRP_{MTB} concentration. The SerC promoter used were of 18 and 20-bp.

Supporting Figure S6

Identical Binding Sites with Cooperativity

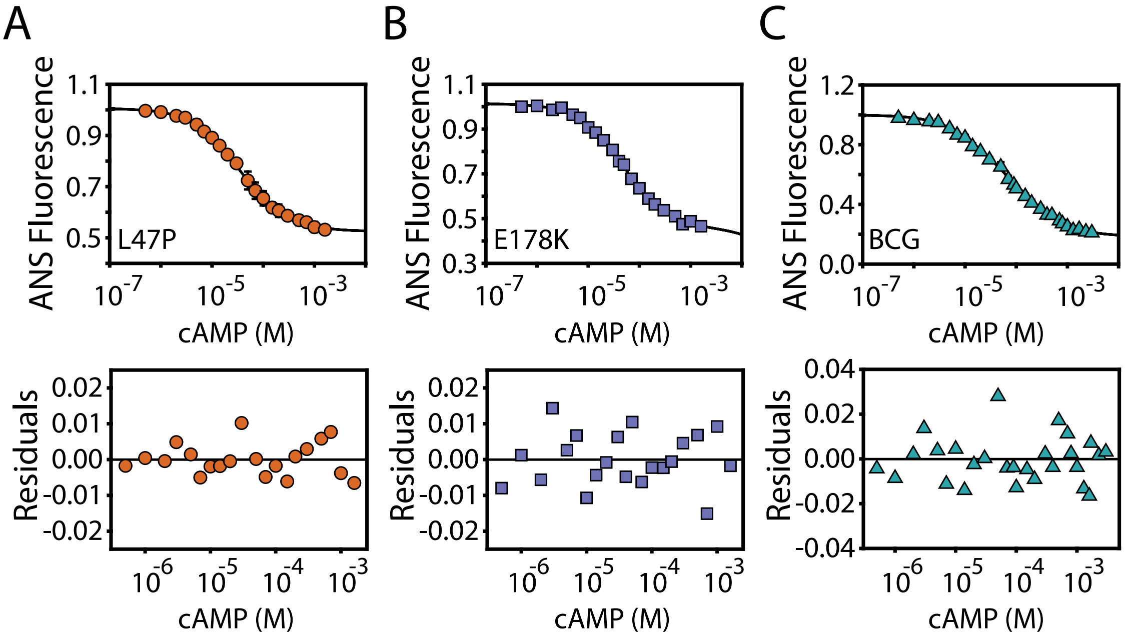

Independent Binding Sites (no cooperativity)

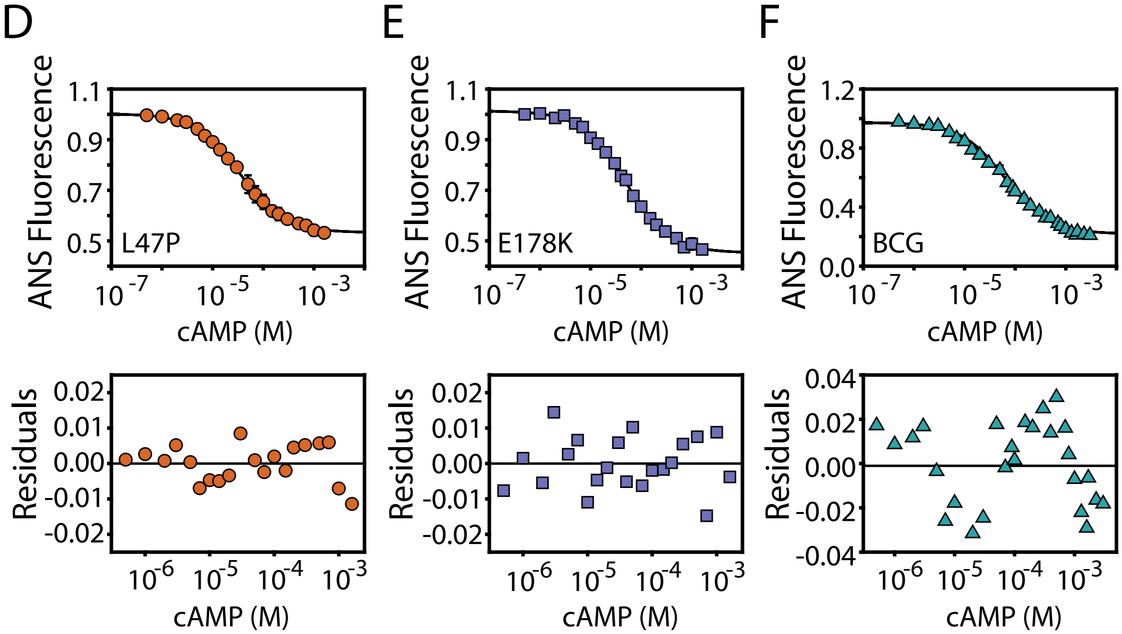

Supporting Figure S6. cAMP binding monitored by changes in ANS fluorescence for CRP mutants. (A-C) The top panels show the ANS fluorescence data and residuals from the fitting of a the two-site binding model with cooperativity. **(D-E)** The bottom two panels display the same ANS fluorescence data and residuals from a fitting of a two-site independent binding site model. Residuals of the fitting are identical for the two models for CRP_{MTB}-L47P and CRP_{MTB}-E178K, indicating that $k_1 = k_2$ (i.e., no cooperativity). However, residuals for CRP_{BCG} show improved random distribution for a model with cooperativity. The binding affinity constants and cooperativities are listed in **Table 4**. Analysis using the *f-ratio* distribution was used to determine which model best fits the data (**Supporting Table S2**).

Supporting Figure S7

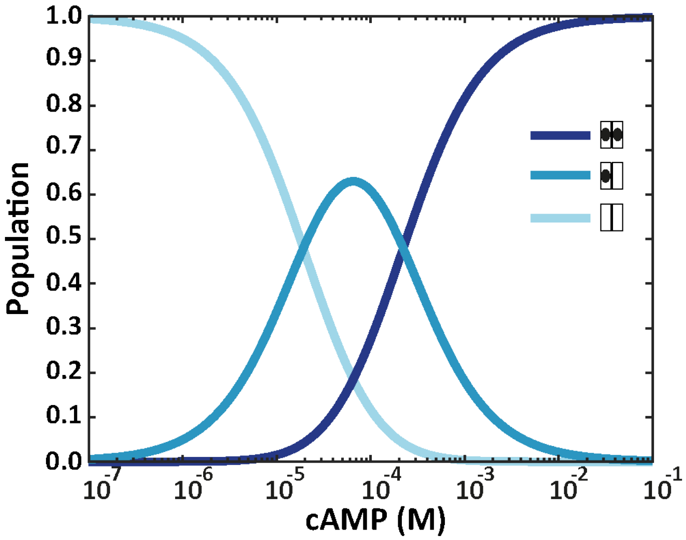

Supporting Figure S7. Simulation of the distribution of populations for CRP_{BCG}. The light blue, blue and dark blue lines correspond to the apo, singly and doubly cAMP-bound populations respectively, simulated using the microscopic binding constants listed in **Table 4**.

Supporting Figure S8

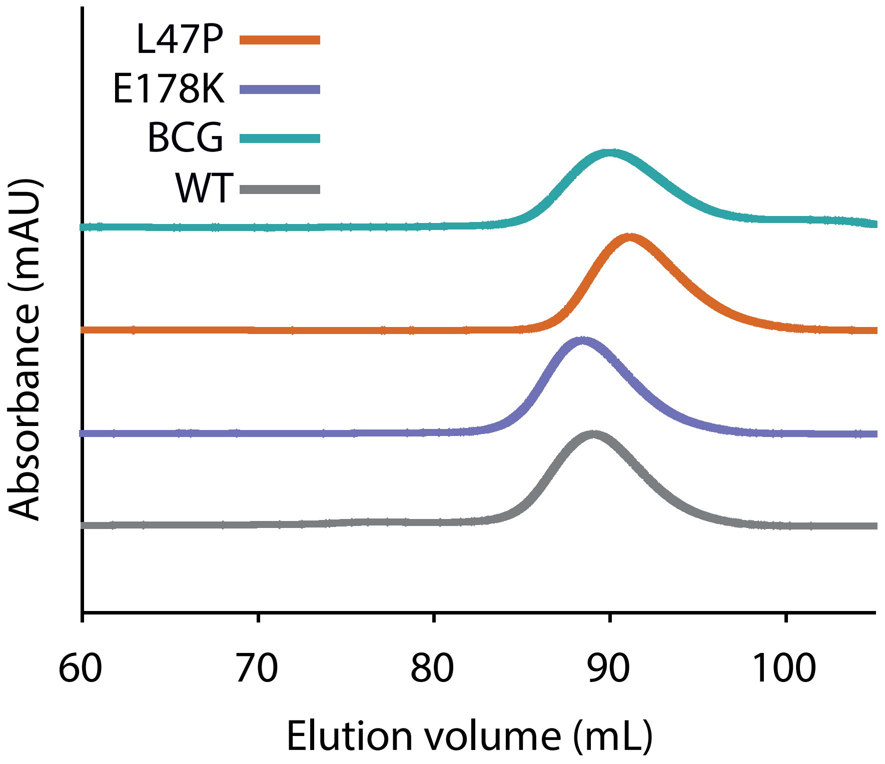

Supporting Figure S8. Size exclusion chromatography elution profiles for CRP proteins. Black, purple, orange, and blue lines correspond to the size exclusion chromatography elution profiles for CRP_{MTB} (wild type), CRP_{MTB}-E178K, CRP_{MTB}-L47P, and CRP_{BCG}, respectively. The elution peak for the proteins is 89.0 ± 2.0 mL for CRP_{MTB} (wild type), 88.4 ± 1.2 mL for CRP_{MTB}-E178K, 91.1 ± 1.6 mL for CRP_{MTB}-L47P, and 90.0 ± 0.9 mL for CRP_{BCG}. The elution peak was obtained from at least three SEC runs.

Supporting Table S1

Supporting Table S1. ITC data fitted to sequential and independent binding sites models

	**Two-site sequential binding sites**						
	n	k_1	k_2	ΔH_1	ΔH_2	ΔS_1	ΔS_2
**HEPES**	2	3.0 ± 0.9	1.2 ± 0.2	5.9 ± 0.6	9.9 ± 1.4	41.6	50.3
**Cacodylate**	2	3.9 ± 0.9	1.8 ± 0.4	4.6 ± 0.5	4.1 ± 0.8	37.4	31.8
**PBS**	2	3.4 ± 0.5	1.2 ± 0.08	5.1 ± 0.2	7.6 ± 0.5	39.0	42.2

	**Two-site independent binding sites**			
	n	k	ΔH	ΔS
**HEPES**	1.6 ± 0.5	2.2 ± 0.08	9.0 ± 0.1	46.1
**Cacodylate**	1.5 ± 0.9	2.6 ± 0.6	6.9 ± 0.2	42.1
**PBS**	1.6 ± 0.4	3.0 ± 0.1	7.4 ± 0.09	44.1

	**Two-site independent binding sites (fixed n = 2)**			
	n	k	ΔH	ΔS
**HEPES**	2	3.0 ± 0.2	6.7 ± 0.2	41.5
**Cacodylate**	2	4.0 ± 0.2	4.8 ± 0.1	35.7
**PBS**	2	4.2 ± 0.4	5.7 ± 0.2	39.0

Error corresponds to standard deviation from fitted parameters as described in Materials and Methods. 'n' corresponds to the number of ligand binding sites. The units of k_1, k_2 and k are 10^4 M^{-1}; ΔH_1, ΔH_2 and ΔH are 10^3 kcal·mol^{-1}; ΔS_1, ΔS_2 and ΔS are in kcal·mol^{-1}·K^{-1}.

Supporting Table S2

Supporting Table S2. Statistical Analysis of cAMP binding data

Isothermal titration calorimetry

	SSR_1	ν_1	SSR_2	ν_2	f_{obs}	C.I.	$P_f > f_{obs}$
HEPES	0.095	19	0.045	17	1.8	90 %	0.06
Cacodylate	0.091	16	0.040	14	2.0	90 %	0.07
PBS	0.187	19	0.020	17	8.4	99 %	$3{\cdot}10^{-5}$

ANS fluorescence

	SSR_1	ν_1	SSR_2	ν_2	f_{obs}	C.I.	$P_f > f_{obs}$
WT	$9.97{\cdot}10^{-4}$	17	$3.71{\cdot}10^{-4}$	16	2.5	96 %	0.02
L47P	$5.24{\cdot}10^{-4}$	16	$3.89{\cdot}10^{-4}$	15	1.3	62 %	0.17
E178K	$11.0{\cdot}10^{-4}$	17	$10.9{\cdot}10^{-4}$	16	0.9	45 %	0.28
BCG	$10.6{\cdot}10^{-3}$	23	$26.9{\cdot}10^{-4}$	22	3.6	99 %	$1{\cdot}10^{-3}$

Subindex 1 indicates binding model 1: identical and independent cAMP binding sites with no cooperativity.
Subindex 2 indicates binding model 2: identical cAMP binding sites with cooperative interactions (i.e., two-site sequential binding sites in **Supporting Table S1**).
C.I. is the confidence interval by which model 1 is statistically equivalent to model 2. In all cases, except for L47P and E178K, cAMP binding models with cooperativity provide a more robust fit with C.I. ≥ 90%.
$P_f > f_{obs}$ is the probability that model 1 provides a better fit than model 2.
